# More Converged, Less Accurate? Reassessing Standard Choices for Ab Initio Water Using Machine Learning Potentials

**DOI:** 10.1021/acs.jpcb.6c02917

**Published:** 2026-07-06

**Authors:** Hubert Beck, Ondrej Marsalek

**Affiliations:** 138735 Charles University, Faculty of Mathematics and Physics, Ke Karlovu 3, 121 16 Prague 2, Czech Republic

## Abstract

Accurately simulating the properties of liquid water remains a central challenge in molecular simulations. In this work, we use machine learning potentials to investigate how the convergence settings of electronic structure calculations impact the predicted structural and dynamical properties of simulated water and ice. We evaluate the true performance of several reference methods in classical and path-integral molecular dynamics. When we compare a popular, computationally pragmatic revPBE0-D3 setup against a highly converged one, our results reveal that its widely reported experimental agreement degrades. Applying the same highly converged settings to the ωB97X-rV functional, we find an improved agreement with experimental results. MP2 with a triple-ζ basis set, commonly used for liquid water, shows poor performance, which is indicative of insufficient convergence. These findings underscore the need for fully converged reference calculations when evaluating the fundamental accuracy of electronic structure methods and developing reliable models for aqueous systems.

## Graphical abstract

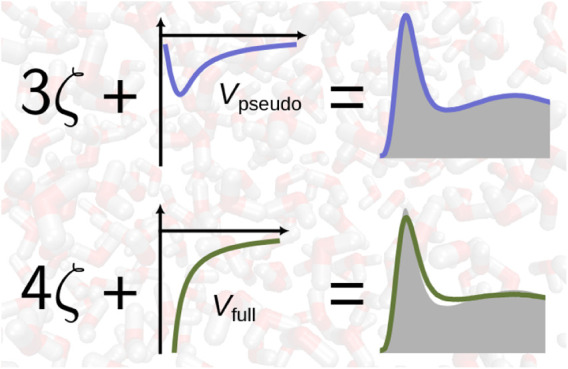

## 1 Introduction

Arguably, there is no other material that has been studied more intensely than water.^1–3^ Despite its simple molecular structure, it has been in the center of attention for many decades and to this date it continues to draw interest and spark debates.^4–6^ Its role as a solvent for many processes, including biological ones,^7^ underscores the importance of finding efficient and reliable ways to simulate the properties of water as accurately as possible. Furthermore, it displays interesting microscopic properties, such as a complex network of hydrogen bonds^8–10^ or intricate dynamics,^11^ as well as macroscopic properties, such as the unique behavior of its density.^12,13^ Accurately modeling experimental properties such as the radial distribution function (RDF), the density, or the diffusion coefficient across the phase diagram is a field of active research.^14–18^

Density functional theory (DFT) has been the main tool in the quest for accurate liquid water properties from first-principles and different levels of theory have been extensively tested.^5,19–22^ In the past decade, machine learning potentials (MLPs) have increasingly gained popularity, driven by their ability to accurately reproduce the potential energy surface (PES) of high-level ab initio electronic structure methods at a substantially lower computational cost.^23,24^ As such, they are perfect for driving long molecular dynamics (MD) simulations and, therefore, have been trained to many different electronic structure methods to find the ideal setup for water.^12,13,17,18,25–27^ These simulations have been used to study the properties of water in its many different phases as well as its interactions with solutes or interfaces.^28–30^

MLPs enabled the study of the importance of including dispersion interactions in water simulations much more thoroughly than with ab initio methods alone.^12,31^ While long-range dispersion interactions are not directly included in most DFT exchange–correlation functionals, they can be added approximately through dispersion correction methods.^32,33^ Some functionals include dispersion interactions directly through a nonlocal correlation term in the functional itself.^34,35^ Alternatively, correlated methods, such as second order Møller–Plesset perturbation theory (MP2), random phase approximation (RPA), or coupled cluster methods, naturally capture long-range electron correlations, including dispersion interactions. Running MD simulations at this level of theory^18,25,26,36,37^ was previously only attainable at enormous computational cost,^38,39^ further highlighting the advantages of MLPs.

With and without the use of MLPs, nuclear quantum effects (NQEs) can be included in MD simulations through imaginary-time path integrals.^40^ NQEs influence many aspects of aqueous systems, such as structure, vibrational properties, or diffusion.^2,18,19,41–44^ Competing NQEs on intermolecular and intramolecular interactions make the resulting properties particularly sensitive to the details of the underlying PES.^11,45,46^ This can result in even seemingly similar models yielding a broad range of outcomes.

One of the most popular exchange–correlation functionals for water simulations is the hybrid functional revPBE0, which is a revPBE generalized gradient approximation (GGA) functional where 25% of the exchange energy was replaced by the exact Hartree–Fock (HF) exchange energy. When evaluated in the TZV2P basis set using pseudopotentials and combined with Grimme’s D3 correction, it reproduces many important experimental findings of water, such as structural properties, the diffusion coefficient, and vibrational spectra, very well.^44^ Therefore, this setup has seen broad adaptation for simulations of liquid water.^17,47–51^ Another promising candidate for simulations of aqueous systems is ωB97X-V,^52^ a range-separated hybrid functional based on the Becke-97 GGA functional^53^ and the VV10 nonlocal correlation functional.^35^ ωB97X-V has been proven to be very reliable across a wide range of benchmarks and shows good agreement with “gold-standard” methods on noncovalent interactions of water molecules.^54–56^ The first post-HF method to be used for bulk liquid water was MP2.^38^ It uses the exact HF exchange, corrected by second order perturbation theory to account for electron correlation effects. Because the computational cost scales as *O*(*N* ^5^) with the number of electrons *N*, the use of MP2 in bulk systems remains limited. Therefore, simulations of bulk water using MP2 have either used a fragment-based approach^9,25,57^ or are constrained in the size of the basis set, even when the simulations are aided by MLPs.^18,38^

When it comes to the benchmarking of methods to reproduce physical observables, most of the attention has been focused on the electronic structure method, dispersion interactions, and NQEs. At the same time, some of the underlying settings of the calculation have not been given sufficient consideration, even though some studies reported relevant differences in RDFs, densities, and diffusion through changes in the electronic structure code, the basis set, or changes in the specifics of the dispersion correction.^13,20,27,38,39,58–60^ Galib et al.^20^ compared water simulated with revPBE0-D3 for different basis sets and found differences in the RDF that were attributed to the parametrization of D3. Del Ben et al.^38,39^ found that including the auxiliary density matrix method (ADMM) approximation in PBE0-D3 simulations leads to a slightly less dense and more structured water. Montero de Hijes et al.^13^ found that the damping function employed in the D3 correction of revPBE0-D3 can impact the temperature–density curve considerably. These underlying parameters play an important role for ab initio MD and will continue to play an important role as MLPs become more and more widespread. However, when ab initio calculations are used to drive MD, basis sets, potentials, and convergence settings have to accommodate the existing constraints in computational resources. Yet, when using the ab initio calculations as reference data to train MLPs, usually the already established setups were used once again without investigating other options. Especially all-electron potentials have rarely been explored for production-level MD simulations of bulk liquid systems using MLPs. Additionally, recent work has shown that many popular databases used to develop MLPs contain a high number of structures with nonzero net forces due to insufficient basis set cutoffs and self-consistent field (SCF) convergence settings.^61^ The advent of MLPs opens the door to routine MD simulations based on high-accuracy methods evaluated with precision, as MLPs’ computational efficiency finally allows us to ignore the fine balance between cost and accuracy and fully commit to obtaining the true PES associated with a certain electronic structure method. Combined with efficient construction of training sets, MLPs can also be used to systematically screen DFT functionals and electronic wave function methods close to the convergence limit based on calculations of macroscopic properties.

In this work, we investigate if common choices for basis sets and potentials are sufficiently converged. We do this by training four committees of neural network potentials (C-NNPs)^62^ on different electronic structure setups and calculating physical observables from MD trajectories obtained with these C-NNPs. Our baseline is a common setup for revPBE0-D3, which is known for an impressive agreement with experimental reference data. We compare this setup with one that uses the same functional and dispersion correction but with a highly converged basis set and an all-electron potential, and one that uses the same tight settings in combination with a ωB97X-rV functional. The fourth model uses a common setup for MP2, a method known for being sensitive to basis set completeness. We examine the role of different convergence settings in the validation of these MLPs. Subsequently, we calculate the radial distribution function (RDF), pressure–density curve, diffusion coefficient, and hydrogen bond lifetime and analyze covalent and hydrogen bonds in detail. Where available, we compare these results with experimental findings. With this meticulous testing scheme, we interrogate whether the impressive agreement of revPBE0-D3 with experiment is due to the excellent performance of the functional or whether some fortuitous cancellation of errors plays a critical role. Our classical and quantum simulations reveal how the changes in the PES induced by practically converged settings play out for thermally averaged static and dynamic properties.

## 2 Computational Details

The electronic structure calculations for training and test sets are executed using the open-source package CP2K^63^ utilizing the Quickstep framework.^64^ In total, four different main setups are used for the calculations, with each of them including dispersion interactions in some form. As our baseline method, we use the revPBE0 hybrid density functional^65–67^ combined with Grimme’s DFT-D3 dispersion correction with zero damping,^68,69^ a setup identical to our previous work.^44,62^ These calculations use the Gaussian and plane waves method^70^ (GPW) in combination with the GTH-PBE pseudopotentials.^71,72^ We employ a robust implementation of HF exchange in periodic systems,^73^ supplementing our primary TZV2P Gaussian basis set with the cpFIT3 auxiliary basis set for the auxiliary density matrix method (ADMM).^74^ The GPW plane-wave cutoff is 400 Ry and the SCF convergence threshold is set to 5 × 10^–7^. We will refer to these settings as revPBE0-D3/TZV2P/GTH. For the revPBE0-D3/def2-QZVP/AE setup, we keep the functional, but tighten several key aspects of the calculations. First, we remove the pseudopotentials and perform an all-electron calculation using the Gaussian and augmented plane waves^75,76^ (GAPW) method instead of GPW. We get close to the complete basis set limit by changing the primary basis set to def2-QZVP and the ADMM auxiliary basis set to def2-TZVP, both introduced by Alrichs.^77^ Furthermore, we increase the plane-wave cutoff to 800 Ry and tighten the SCF convergence threshold by 2 orders of magnitude to 5 × 10^–9^. To better analyze the separate roles that the potential and basis set play, we also create a revPBE0-D3/def2-QZVP/GTH setup that is identical to revPBE0-D3/def2-QZVP/AE, but uses the GTH pseudopotentials instead of all-electron potentials. For the ωB97X-rV/def2-QZVP/AE setup, we keep these improved convergence settings, but replace the revPBE0-D3 functional with the ωB97X-rV^52^ range-separated hybrid functional. To include dispersion interactions, it uses the revised VV10 nonlocal correlation functional^78,79^ (indicated by the “–rV”), an adaptation of the VV10 functional^35^ optimized for evaluation in plane wave basis sets, with parameters *b* = 6.0 and *C* = 0.01.

The final setup, denoted MP2/cc-TZ/GTH, uses second order Møller–Plesset perturbation theory^80^ to treat electronic correlation explicitly. Unfortunately, running all-electron calculations of bulk structures close to the basis set limit for MP2 would be prohibitively expensive for our geometries, even for single-point calculations. Hence, we use the Resolution of Identity Gaussian and Plane Wave^81–83^ (RI-GPW) approach with GTH pseudopotentials optimized for HF theory in combination with a correlation-consistent triple-ζ (cc-TZ) basis set^82,84^ and an auxiliary triple-ζ basis set for the resolution of identity calculations.^82,85^ We set the general plane-wave cutoff to 800 Ry and the cutoff for calculating MP2-integrals to 300 Ry. The SCF convergence criterion is set to 10^–6^. This setup has also been used by the first studies of bulk liquid water with MP2 ^38^ as well as one study using MLPs,^18^ which will become useful to compare results.

As an example of computational demands of the different methods, we can examine the run time and peak memory utilization with the different setups for 64 bulk water molecules. To accelerate SCF convergence, we use converged Kohn–Sham orbitals from the GGA counterpart to initialize each hybrid functional’s SCF procedure, and PBE orbitals to initialize the HF SCF in the RI-MP2 calculation. A single-point calculation with the original revPBE0-D3/TZV2P/GTH method on a node with 2 AMD EPYC 7301 16-core CPUs took 4 min and required 18 GB of memory. A single-point calculation on the same hardware took roughly 14 min for revPBE0-D3/def2-QZVP/AE and 34 min for ωB97X-rV/def2-QZVP/AE and required 35 GB and 123 GB of memory, respectively. The MP2/cc-TZ/GTH calculations were run on 16 nodes with 2 AMD EPYC 7H12 64-core CPUs each, and a single point calculation took ca. 28 min with a memory requirement of 3.2 TB. Further details on the resource requirements can be found in the Supporting Information S2.

The training data comprises a total of 964 structures from different aqueous systems, which were selected from MD trajectories using the query by committee (QbC) process.^62^ We extended the 814-structure canonical ensemble (*NVT*) water data set by Schran et al.^62^ with 150 additional structures sampled from MD simulations in the isobaric–isothermal ensemble (*NpT*) to improve the density prediction of the model. Further details can be found in Section S1. The data set now contains bulk structures of liquid water (64 molecules), ice I_h_ (96 molecules), ice VIII (64 molecules), as well as a water slab (216 molecules), taken from classical and path integral MD (PIMD) simulations at different temperatures and densities. Energies and forces are evaluated for these geometries at the different levels of electronic structure theory that we introduced above to create the four training sets. Unfortunately, the memory requirements of a 216-molecule slab surpass our available resources, so the MP2 training set is limited to liquid water and bulk ice structures.

The C-NNPs consists of 8 separate Behler–Parrinello deep neural networks^23,86^ as implemented in the N2P2 package.^87,88^ Each committee member is trained on a different 90% subset of the total training data set and initialized with different weights following the Nguyen–Widrow scheme^89^ at the start of each training procedure. The mean over all committee members forms the eventual PES prediction. The NNPs are trained on both energies and forces using a multistream adaptive extended Kalman filter,^90,91^ and the remaining training settings follow the recommendations for water from the N2P2 developers. The NNPs are trained for 1000 epochs, substantially longer than in previous work, as our tests have shown no signs of overtraining even for such a high number of epochs but instead a slight improvement of predictions on an independent test set. As input features, we use atom-centered symmetry functions^86^ (ACSF) with 32 pairwise functions (8 functions for each combination of hydrogen and oxygen atoms) and 25 angular symmetry functions.^12^ Before training, each feature is preprocessed by subtracting its mean across the whole data set and scaling the values to cover a range of 1.

With these four C-NNPs, we run classical and path integral MD and simulations^40^ in both the *NVT* and *NpT* ensemble. These simulations are performed using the i-PI package^92^ with interactions provided by CP2K,^63^ which has an efficient implementation of committees of Behler–Parrinello potentials.^62^ The simulation boxes for liquid water contain 512 molecules, with a 24.84 Å cubic box in *NVT* calculations, corresponding to the experimental density of liquid water at ambient conditions. For ice I_h_, we use a system of 96 molecules in a box of dimensions 13.489 × 15.576 × 14.641 Å, again in accordance with experimental results. The temperatures are set to 300 K and 250 K for liquid water and ice I_h_, respectively. The production *NVT* trajectories of bulk liquid water have a length of 1 ns with a 0.5 fs time step for classical MD, and a length of 250 ps with a 0.25 fs time step for PIMD. The *NpT* simulations have a length of 100 ps at each pressure. In classical MD simulations, the temperature is controlled by a velocity rescaling thermostat^93^ with a time constant τ of 1 ps. For the path integral simulations, we perform thermostated ring polymer molecular dynamics (TRPMD)^94,95^ with 32 replicas, employing a global path integral Langevin equation (PILE-G) thermostat^96^ with τ = 1 ps and λ_PILE_ = 0.5. The *NpT* simulations are run in an isotropically scaling cell at pressures of 1, 1000, 2000, 3000, 4000, and 5000 atm for classical simulations and 1, 2000, and 5000 atm for PIMD. The barostat is a Bussi–Zykova–Parrinello barostat^97^ with a time constant of 200 fs, thermostated by a smart-sampling generalized Langevin equation (GLE) thermostat.^98,99^

Moving on to dynamical properties, the diffusion coefficient *D* can be expressed in terms of the mean square deviation MSD using Einstein’s relation:

1 $$D=\frac{\mathrm{MSD}}{6t}=\frac{1}{N}\underset{i}{\sum }\frac{\left\langle {|r_{i}(0)-r_{i}(t)|}^{2}\right\rangle }{6t}$$

where **r**_*i*_(*t*) are the positions of *N* atoms at time *t*. To correct for finite-size effects in a periodic cell, we add the correction term^100^

2 $$D_{\mathrm{corr}}=\frac{k_{B}T\xi }{6\pi \eta }\frac{1}{L}$$

where *k*_B_ is the Boltzmann constant, *T* is the temperature, η is the shear viscosity of the liquid, and ξ = 2.83729 accounts for the cubic shape of the cell of length *L*. We use the experimental value η = 0.8925 mPa s for water at 300 K for all our models. This is only an estimate of the true correction factor, which would use the shear viscosity of each model. As an alternative, extrapolation to an infinite cell size based on simulations at multiple different system sizes could also be used.^12^

The hydrogen bond lifetimes are calculated from the hydrogen bond existence criteria *h*_*n*_(*t*)­

3 $$h_{n}\left(t\right)=\Theta \left(r_{n}\left(t\right)-r_{0}\right)\Theta \left(\theta_{n}\left(t\right)-\theta_{0}\right)$$

where Θ is the step function and *n* is an index that runs over all potential hydrogen bonds in the system. *r*_*n*_(*t*) is the distance between the two oxygen atoms and θ_*n*_(*t*) is the angle between the vector connecting the two oxygen atoms and the vector connecting the donor oxygen and its covalently bonded hydrogen atom at time *t*. We use the cutoffs *r*_0_ = 3.5 Å and θ_0_ = 30° for the distance and angle, respectively.^101,102^ For path integral trajectories, we use the RPMD average over the *P* replicas and thus have

4 $$h_{n}\left(t\right)=\frac{1}{P}\overset{P}{\underset{j=1}{\sum }}\Theta \left(r_{n}^{\left(j\right)}\left(t\right)-r_{0}\right)\Theta \left(\theta_{n}^{\left(j\right)}\left(t\right)-\theta_{0}\right)$$

where $r_{n}^{\left(j\right)}$ and $\theta_{n}^{\left(j\right)}$ are the distance and angle for replica *j*. This *h*_*n*_(*t*) can now take fractional values between 0 and 1 with a denominator *P*. We calculate the autocorrelation function of *h*_*n*_(*t*) averaged over all *N*_HB_ hydrogen bonds that occur in the trajectory as

5 $$C\left(\tau \right)=\frac{1}{N_{\mathrm{HB}}}\overset{N_{\mathrm{HB}}}{\underset{n=1}{\sum }}\frac{{\langle h_{n}\left(t_{0}\right)h_{n}\left(t_{0}+\tau \right)\rangle }_{t_{0}}}{\left\langle h_{n}\right\rangle }$$

which gives the probability that a hydrogen bond that existed at time *t*_0_ exists at time *t*_0_ + τ.^102,103^ Finally, we integrate *C*(τ) over τ to obtain a time that characterizes the lifetime of the hydrogen bond.

## 3 Results

We will start by inspecting the four C-NNPs and validating that all of them reliably reproduce the PES of their reference method. Next, we will calculate physical observables from the trajectories obtained with these models. By comparing them with each other and with experimental reference data, we will elucidate the role that pseudopotentials, basis sets, and exchange–correlation functionals play in the simulation of water.

### 3.1 Model Validation

Comparing the predictions of a model to a reference data set independent from the training data set is considered the gold standard for validating machine learning models. For this purpose, we use the test set introduced by Schran et al.^62^ The test set was initially constructed to validate the performance of the C-NNP in comparison to ab initio calculation with the revPBE0-D3/TZV2P/GTH setup. We made some modifications to address the increased computational demands of our highly converged setups and MP2 calculations, as well as the inclusion of *NpT* simulations. The test set consists of 500 structures sampled from each classical MD and PIMD simulations in the *NVT* ensemble of bulk liquid water and ice I_h_, as well as 200 structures from each classical and path integral MD of a slab of liquid water. Since we will use the MLPs to calculate pressure–density curves, we also added structures sampled from *NpT* simulations. For this test set, we sampled 100 structures each from *NpT* simulations at 1, 1000, 2000, 3500, and 5000 atm to obtain a total of 500 structures. For the MP2 test set, it was necessary to reduce the number of structures to 100 each from classical and quantum simulations of *NVT* and *NpT* trajectories at 300 K. Furthermore, no ice structures are included in the MP2 test set, as these have been shown to score the best out of all systems for every model. Therefore, we do not expect negative outliers in this phase. The force root-mean-square errors (RMSEs) of a selection of these test sets are shown in the top panels of Figure 1, while the remaining test sets as well as the energy errors can be found in Figure S2. The errors for all models across all test sets are low enough to reliably reproduce the static and dynamic properties of the ab initio reference methods.^17,28,62^ As expected, the errors for ice are lower than for liquid water due to the narrower exploration of the configuration space. Meanwhile, the errors for the slab test sets are higher because the molecules at the liquid–vapor interface are more challenging for the model, and there is also less reference data for them in the training set. Surprisingly, the errors of the models trained on the recalculated data are even lower than for the original model. This, however, is not primarily a result of more accurate predictions, but of inaccuracies in the revPBE0-D3/TZV2P/GTH test sets. Due to the so-called “egg box effect”,^104^ which is a major source of error in the GPW calculations of revPBE0-D3/TZV2P/GTH, but suppressed in the GAPW and MP2 calculations, all revPBE0-D3/TZV2P/GTH data sets suffer from a small amount of additional noise. During training, the NNPs are not capable of learning the egg box effect, which depends on absolute atomic positions, because the ACSFs depend only on relative atomic positions. Therefore, the egg box effect adds aleatoric uncertainty to the training data. The models will smooth over most of this added noise, but the egg-box variation in test-set reference calculations contributes to the error when evaluating the performance of a model. Recently, Kuryla et al.^61^ made similar observations when they discovered a high number of structures with nonzero net forces in many popular databases used to develop MLPs. These net forces indicate errors in the reference calculations caused by inadequate SCF settings and insufficient basis sets. They defined a net force above 1 meV/Å/atom as the threshold for problematic structures. We found substantial net forces for our revPBE0-D3/TZV2P/GTH data set—an average of 2.14 meV/Å/atom over all its structures. For the remaining data sets, the net forces were significantly lower—0.08 meV/Å/atom for both revPBE0-D3/def2-QZVP/AE and ωB97X-rV/def2-QZVP/AE and 0.002 meV/Å/atom for MP2/cc-TZ/GTH. Having identified this issue, Kuryla et al. attempted to correct calculation settings for many popular electronic structure codes. However, for CP2K, a considerable nonzero net force still remained after tightening many key settings. We show that switching from the GPW to the GAPW method helps reduce the remaining net forces considerably. We recalculated the “LW NpT” set with the revPBE0-D3/TZV2P/GTH settings, but using the GAPW method and a higher plane wave cutoff of 800 Ry and obtained net forces of 0.07 meV/Å/atom and test set errors similar to the other GAPW methods. This shows that GAPW’s suppression of the egg-box effect^75^ is an important tool for reducing inconsistencies in CP2K calculations. Further insights into this issue and its effect on training, including for modern high-capacity models, can be found in Section S6.

**Figure 1:**
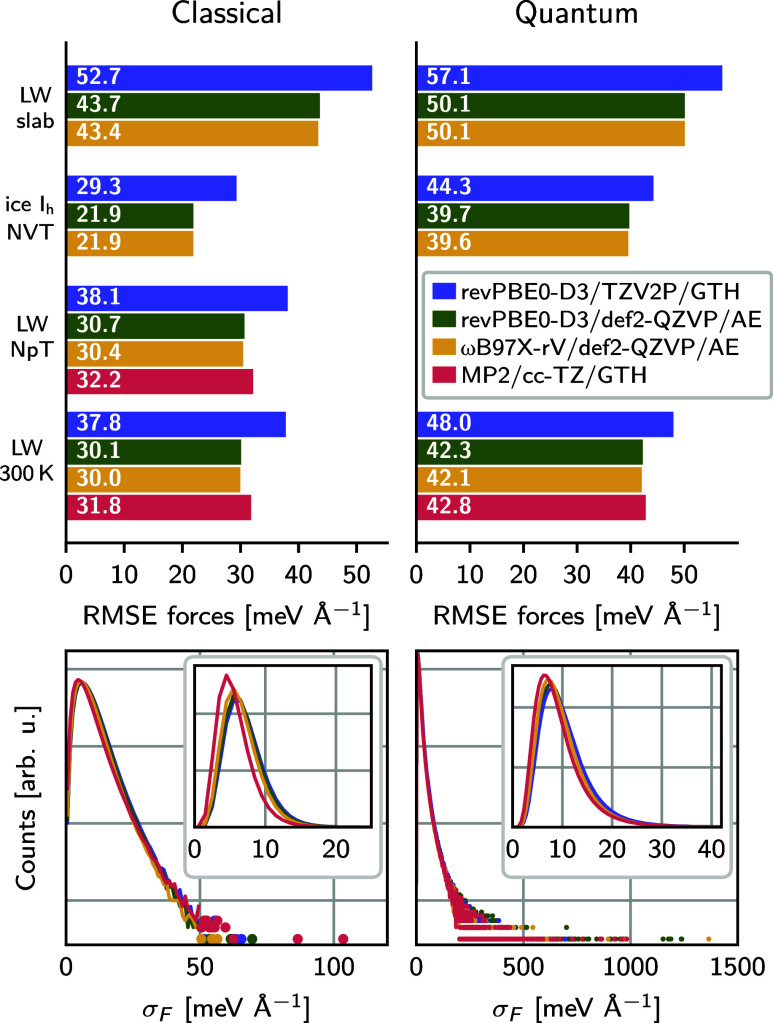
Top panels: RMSEs for the 4 different C-NNPs on test sets of liquid water, ice and a slab with a liquid–vacuum interface for both classical (left) and quantum (right) structures. Bottom panels: Distribution of force component disagreements *σ_F_* along an *NVT* trajectory of liquid water using classical (left) and path integral (right) MD. The main panels use a logarithmic scale for the *y*-axis, while the insets shows the distribution for low disagreements on a linear scale.

Since the different reference methods also change the structures sampled by MD, we compared the distribution of committee disagreement in MD simulations using the established revPBE0-D3/TZV2P/GTH C-NNP to those using the models trained on the new reference methods. The distributions are displayed in the bottom panels of Figure 1. They are very similar for all four C-NNPs, indicating reliable predictions. For path integral simulations, we find a number of structures with noticeably high (>500 meV Å^–1^) force disagreements. However, as the inset in the bottom right panel emphasizes, these cases represent a negligible fraction of the total data and therefore do not negatively impact the final results. These committee disagreements in combination with the low test set errors prove that recalculating energies and forces for the geometries of the original training set is a viable strategy to obtain a reliable C-NNP for a different reference method. In principle, the model for the new method could show an appreciable increase in disagreements during MD or in errors evaluated on a test set. In that case, one could simply continue the QbC-based active learning that created the original training set to include additional structures required to make the new PES well-determined by the data.

### 3.2 MD Simulations

We will now turn our focus to analyzing the MD trajectories. With the exception of the pressure–density analysis, we always use *NVT* simulations of bulk liquid water and keep a consistent color scheme for all plots: revPBE0-D3/TZV2P/GTH is shown in blue, revPBE0-D3/def2-QZVP/AE in green, ωB97X-rV/def2-QZVP/AE in yellow, and MP2/cc-TZ/GTH in red. Results are shown as solid lines/bars for classical MD and as dashed lines/bars for PIMD.

We start the investigation of structural properties of liquid water by examining the oxygen–oxygen (O–O) radial distribution function (RDF) shown in Figure 2. It has been shown in previous studies^44^ that classical revPBE0-D3/TZV2P/GTH matches the experimental results^105^ closely. Adding NQEs improves the result even further by slightly shortening the onset of the first coordination shell and by deepening the first minimum. Unfortunately, the fully converged revPBE0-D3/def2-QZVP/AE setup shows that this immaculate match to the experimental benchmark is in part enabled by the incomplete convergence of the original computational setup. The converged RDF features a flatter peak at the first hydration shell and a mostly flat RDF beyond that. As can be seen in Figure S3, the main difference in the RDF stems from the change in basis set, whereas the all-electron potential contributes only negligibly. It should be noted that in addition to this highly converged primary basis set, we also switch to a considerably richer ADMM auxiliary basis set. Del Ben et al. suggests in a study using PBE0 that a small ADMM basis set can lead to an increased structure in the O–O RDF.^38,39^ NQEs slightly improve the results, but the difference from the experimental findings is still substantial. Galib et al. have run calculations for revPBE, the GGA counterpart of revPBE0, with the TZV2P and MOLOPT-DZVP-SR-GTH basis sets and found that without dispersion correction the O–O RDFs for both setups matched well, but including D3 dispersion corrections lead to substantial differences.^20^ They attributed this result to the D3 parametrization and its basis set. ωB97X-rV/def2-QZVP/AE reproduces the key features of the experimental results moderately better, though a considerable error remains. A study by Yao et al. used ωB97X-rV with the TZV2P basis set and GTH pseudopotentials and reported an overstructured O–O RDF.^16^ This confirms the trend of a larger basis set loosening the water structure we observed for revPBE0-D3. While the RDFs of all three DFT sets clearly resemble the experimental one, MP2/cc-TZ/GTH is a strong outlier. Overall, the RDF is severely overstructured, with the peak of the first coordination shell being way too high and at a too short distance. Subsequent peaks are too high and valleys are too deep. These findings for MP2 agree well with the original ab initio MD findings^38,39^ and the more recent MLP simulations using the same setup for the electronic structure reference calculations.^18^ They attributed this behavior to an overestimation of binding energies due to the small basis set. Other studies using a fragment-based MP2 approach in combination with a high-quality basis set obtained a better fitting RDF.^9,25,57^ Dào et al.^27^ used the cc-TZ basis in combination with revPBE0-D3 and found that it leads to an overstructuring of the RDF. Although we should not draw any direct conclusions from these hybrid DFT results for MP2 calculations using the same basis set, it further emphasizes how the basis set impacts the structural properties.

**Figure 2:**
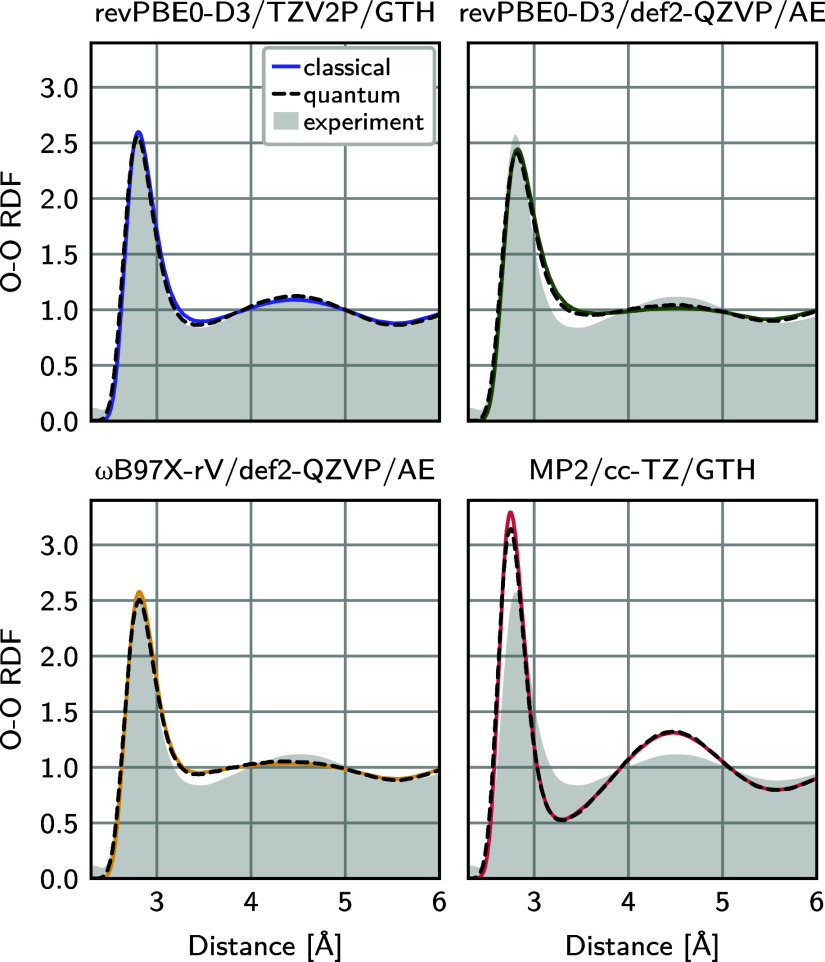
Oxygen–oxygen RDF for the 4 C-NNPs. In each panel, the result from the classical MD simulation is shown by the solid line in the respective color, the PIMD result is shown by the black dashed line and the experimental reference^105^ is indicated by the gray-shaded area.

For further insights, we will look closer at the structure of the water molecules and the interactions with the first coordination shell. As shown in the top panel of Figure 3 and the inset bar chart, the changes in the reference method have only a small effect on the lengths of the covalent bonds. For classical MD, the means of all 4 distributions lie within less than 0.01 Å, or less than 1% of the total bond lengths, with revPBE0-D3/def2-QZVP/AE having the shortest covalent bonds and MP2/cc-TZ/GTH the longest. NQEs lead to much wider distributions, whose means are moderately longer than those for classical nuclei. The order of the ab initio methods remains the same and the difference between them remains almost perfectly constant. Tests with the revPBE0-D3/def2-QZVP/GTH setup (see Figure S4) suggest that the changes in basis set and potential result in opposite outcomes, effectively canceling each other out. The middle panel of Figure 3 shows the proton sharing coordinate $\delta =d_{\mathrm{HO}}-d_{{\mathrm{HO}}^{\prime }}$ , which measures the difference of the distances between the hydrogen atom and the donor and acceptor oxygen atoms. Quantum delocalization shifts the distribution toward protons being shared more equally between the two oxygen atoms. In rare cases (0.0015% to 0.0045%), this even results in the so-called “proton excursion”, where the proton is closer to the acceptor atom than the donor atom, i.e., δ > 0. None such cases can be observed without NQEs. These numbers for revPBE0-D3/TZV2P/GTH agree well with previous findings.^44^ Comparing the methods, a split between the DFT functionals and MP2 is apparent. The MP2 trajectories exhibit a shift toward more shared protons, as well as flatter distributions at low δ and more pronounced peaks. The bottom panel of Figure 3 shows the angle between the donor–acceptor vector and the covalent bond vector. NQEs generally shift the distributions toward wider angles compared to their classical counterparts, while the distribution becomes flatter. When comparing the DFT setups, we see that the highly converged revPBE0-D3 shows a wider distribution compared to the standard settings, with ωB97X-rV being close to revPBE0-D3/def2-QZVP/AE. The half width at half-maximum (HWHM) of the distributions serves as a good measure for fluctuations in the HB angle, which are related to the strength of the hydrogen bonds^12^—a broader distribution corresponds to weaker hydrogen bonds. Furthermore, for all our models, we find consistently smaller HWHM than Morawietz et al., who employed GGA functionals with and without dispersion corrections.^12^ As with the δ coordinate, the differences between MP2 and the DFT functionals are considerable. The MP2 peaks are shifted toward sharper angles and the tail of the distribution above 25° is strongly suppressed.

**Figure 3:**
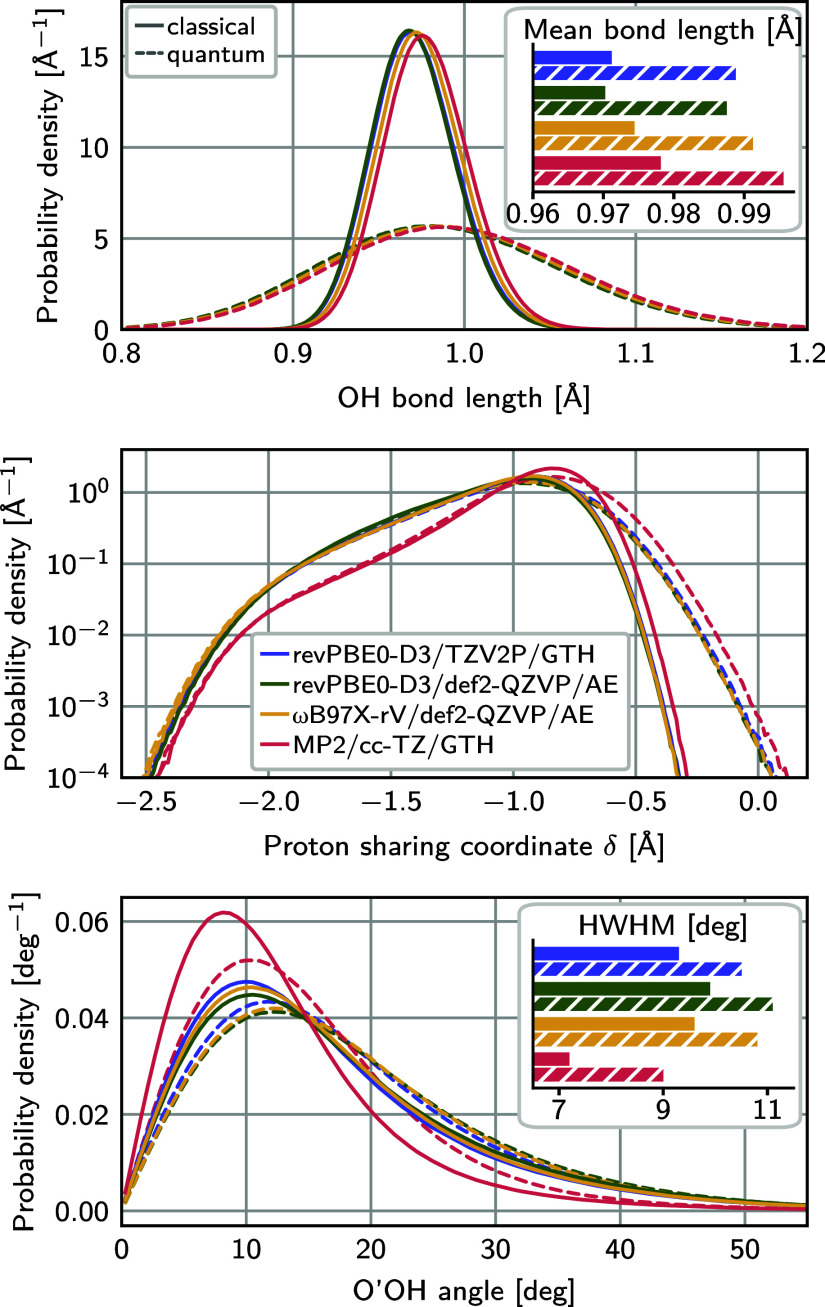
Analysis of the structure of covalent and hydrogen bonds. Top panel: the distribution and means of lengths of covalent oxygen–hydrogen bonds for classical (solid lines/bars) and PI (dashed lines/bars) MD run with the 4 different C-NNPs. The main plot shows the distributions of bond lengths and the inset the mean bond lengths. Middle panel: The distributions of the proton sharing coordinate. Bottom panel: The distributions of the hydrogen bond angle. The inset compares the half width at half-maximum of the different methods.

One of the most intensely debated topics in ab initio and MLP water simulations is the density.^12,13,20,31,45,59,106,107^ Therefore, we analyze *NpT* trajectories and test how well the different methods can reproduce the density at different pressures. Figure 4 shows the pressure–density curve for water (top panel) and ice I_h_ (bottom panel) for pressures between 1 and 5000 atm and compares them with experimental results^108^ (indicated in black). It shows how the established setup revPBE0-D3/TZV2P/GTH underestimates the density of both liquid water and ice. Our results agree well with a study by Cheng et al., who used an MLP trained on the same reference method but with a training set constructed in a way different from ours.^17^ Upgrading the basis set has a considerable impact on the density of liquid water, giving us a curve that closely matches the experimental curve, especially for low densities. The high sensitivity of the pressure to the basis set has been remarked previously in the context of plane wave basis sets.^107^ In our case, the improvement comes from the increased flexibility of the primary Gaussian basis set used for the Kohn–Sham orbitals. The density given by these orbitals then needs to be expanded in the auxiliary basis set, which in the case of the GAPW method is a combination of plane waves and local corrections based on the primitive Gaussians of the primary basis set. On the other hand, tests with pseudopotentials presented in Figure S5 show that the impact of the potential on the density is negligible. Although the density does not increase fast enough with increasing pressure, it is still in very good agreement with the experimental results. Willow et al. showed in tests with parametrizable force fields that polarizability is an important factor in determining at which pressure water has a density of 1.000 g cm^–3^.^14^ A higher polarizability, which is usually associated with larger basis sets,^14,109–111^ corresponds to a lower pressure at which experimental ambient density is reached.^14^ The ADMM basis set and the interplay between dispersion correction and basis set, both of which we have already discussed for the RDF, likely play a role here as well.^20,38^ A study using revPBE0-D3 in a highly converged plane wave setup obtained an even higher density of roughly 1.03 g cm^–3^.^13^ For ice I_h_, however, both revPBE0-D3 models give almost identical results. ωB97X-rV/def2-QZVP/AE overestimates the density of liquid water across the whole range of pressures, although more strongly for low pressures than for high pressures. For ice I_h_, it reproduces the experimental reference point^112^ the best out of all the models, especially when including NQEs. Despite the unsatisfactory RDF, MP2/cc-TZ/GTH gives a decent pressure density curve for liquid water. Even though it is about 0.02 g cm^–3^ too low, it replicates the overall shape of the experimental result better than our other setups. It is also in decent agreement with ab initio results, considering their limited number of Monte Carlo cycles.^38^ On the other hand, MP2 overestimates the experimental density for ice I_h_. As for all models, the density of ice increases linearly with pressure while being lower than that of liquid water. When comparing classical and path integral simulations of liquid water and ice, we see an increase in density between 0.005 and 0.011 g cm^–3^ in most cases when including NQEs. The two exceptions are liquid water with the revPBE0-D3 models, where we observe no NQEs on the density outside the error bars. This is in contrast to a density increase due to NQEs reported in the above-mentioned ref 17.

**Figure 4:**
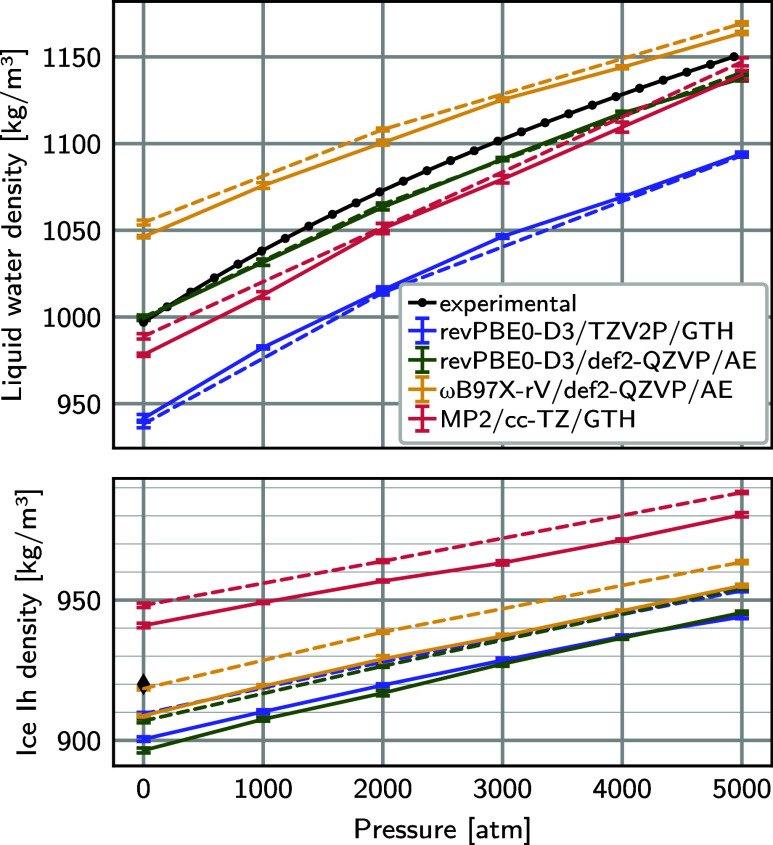
Pressure–density curve of liquid water (top) and ice I_h_ (bottom) for the 4 different C-NNPs. The black curve in the top panel and the black diamond in the bottom panel shows the experimental reference for each system.^108,112^ The error bars indicate statistical errors obtained by block averaging. As in the other plots, solid lines are used for classical MD and dashed lines for PIMD.

Let us now take a look at dynamic properties, starting with a comparison of the diffusion coefficient for the different methods. Figure 5 shows the diffusion coefficients we obtained and compares them with the experimental value of (2.41 ± 0.15) × 10^–9^ m^2^ s^–1^.^113^ The lightly shaded area of the bars indicates the contribution from the correction we applied to account for the finite size of the simulation cell. Our results for revPBE0-D3/TZV2P/GTH match ab initio findings (corrected at a different cell size) adequately,^44^ which further validates the MLPs. Both classical and path integral simulations give a diffusion coefficient within the confidence interval of the experiment, with a decrease of 8% due to NQEs. However, analogously to what we have already seen with the RDF, using a more complete basis set as well as all-electron potentials leads to less structure in the system and increases diffusion by 0.9 and 0.95 × 10^–9^ m^2^ s^–1^ for classical and path integral simulations, respectively. A comparison of the three revPBE0-D3 models in Figure S6 shows that the larger basis set and the all-electron potential both result in faster diffusion, with the basis set having a stronger impact than the potential. ωB97X-rV/def2-QZVP/AE gives a diffusion coefficient closer to the experimental result, though still somewhat overestimated. Furthermore, in contrast to revPBE0-D3 simulations, including NQEs results only in a negligible increase in diffusion. Clear problems become apparent for the MP2/cc-TZ/GTH setup, where the diffusion is severely underestimated. Notably, a substantial part of the total diffusion coefficient does not even originate from actual diffusion in the simulation, but rather from the finite-size correction of 0.28 × 10^–9^ m^2^ s^–1^ (36% of the total). Furthermore, we observe a modest increase in diffusion when NQEs are included. For comparison, with explicit MP2 AIMD at the classical level, Del Ben et al.^39^ report 0.67 × 10^–9^ m^2^ s^–1^ without finite size corrections in a smaller system of 64 molecules and with much shorter trajectories (2 × 10 ps). MLP simulations by Li et al.^18^ referencing the same MP2 setup obtained 0.693 × 10^–9^ m^2^ s^–1^, including a finite size correction based in the experimental viscosity that is a factor of 4 smaller than ours, despite their simulation cell being smaller. They also report a larger difference between classical and quantum simulations (1.060 × 10^–9^ m^2^ s^–1^ for PIMD) than our observations. It is not straightforward to determine how these differences in simulations with what should nominally be the same PES emerge as a result in the specifics of these different studies. The fragment-based MP2 approach used by Liu et al. reports 1.80 × 10^–9^ m^2^ s^–1^ for classical MD and 2.27 × 10^–9^ m^2^ s^–1^ for PIMD.^25^ Here, the PES itself is different due to a difference in the basis set and the use of fragmentation, so a strict match should not be expected. Further investigation would be required to determine whether this improved performance is indeed due to the denser basis set or due to some cancellation of errors stemming from the MP2 method and the fragment-based approach.

**Figure 5:**
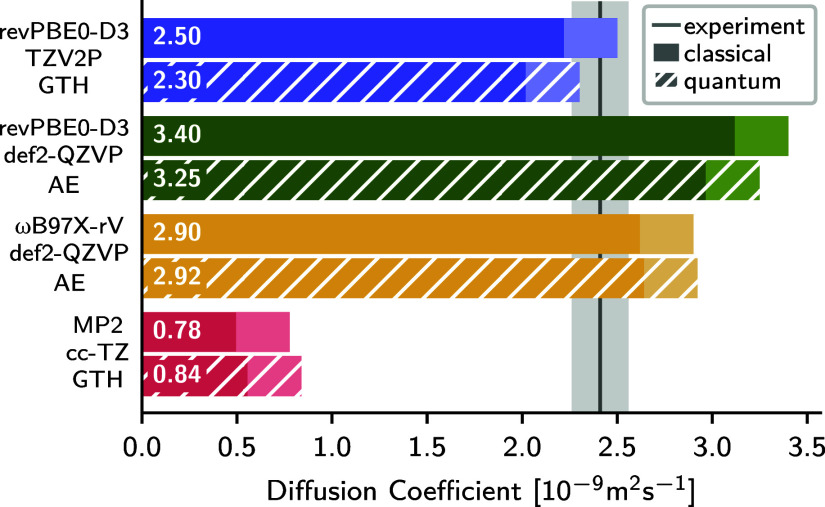
Comparison of the diffusion coefficients for the different C-NNPs in classical and quantum MD. The pale colors at the end of each bar signal the magnitude of the finite-size corrections added to the calculated values. The gray vertical area indicates the experimental reference value including its confidence interval.^113^

We further investigate the origins of these differences in the dynamics by looking at the characteristic lifetimes of hydrogen bonds in each trajectory. The results of these calculations are shown in Figure 6. There is a strong anticorrelation between diffusion coefficients and hydrogen bond lifetimes, as could be expected. Strong hydrogen bonds have longer lifetimes, which corresponds to slower diffusion. Equivalently, weak diffusion means that a broken hydrogen bond has a higher chance to recombine again, as the two molecules remain close to each other.^102^ Compared to the established revPBE0-D3 setup, the larger basis set and the switch to an all-electron potential (further details in Figure S7) in revPBE0-D3/def2-QZVP/AE result in shorter-lived hydrogen bonds. Similar to the results for the diffusion, the consequences of the basis set change are stronger than for the potential. With the same setup, the ωB97X-rV functional yields roughly 20% higher lifetimes. For all three DFT setups, including NQEs increases the lifetimes, with ωB97X-rV showing the smallest effect. For MP2/cc-TZ/GTH, we find very long lifetimes of around 20 ps with a quantum effect that goes in the opposite direction, decreasing the time.

**Figure 6:**
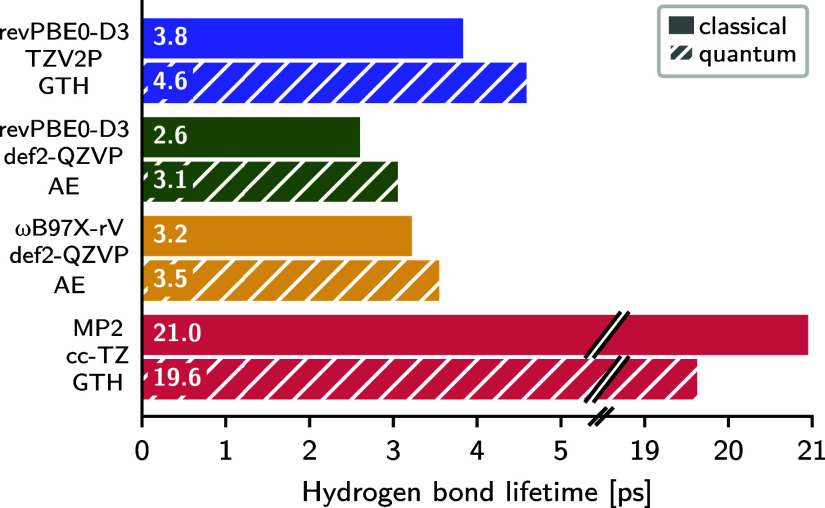
Hydrogen bond lifetimes for the 4 different C-NNPs. To allow for an easier analysis of the shorter bars, we made a cut in the *x*-axis between 6 and 18 ps, indicated by the double-dashes in the axis and the affected bars.

Through the lens of competing NQEs on hydrogen bonding,^45^ we can see how the changes in the PESs due to more tightly converged settings or differences between functionals manifest in the resulting properties of water. The geometry of the hydrogen bonds shows both their strengthening due to the intramolecular NQE and their weakening due the intermolecular NQE, with the compensation being similar across the DFT methods. For MP2, the intramolecular NQE is more pronounced. The net NQE is then reflected in diffusion and hydrogen bond kinetics. For revPBE0-D3, there is modest slowdown, with the tighter settings yielding a somewhat smaller effect. For ωB97X-rV/def2-QZVP/AE, the effect is the smallest, indicating near-complete compensation. MP2 shows the opposite NQE, with both diffusion and hydrogen bond decay speeding up. This result should be interpreted with caution, though, due to how overstructured and slow MP2 water is, at least with these settings.

### 3.3 Discussion

The changes in static and dynamic physical observables discussed above are expressions of changes in the PES of the underlying method. However, as Figure 7 illustrates, it can be difficult to quantify how differences between various PESs affect physical observables. There, we plot the distribution of absolute differences of force components between revPBE0-D3/TZV2P/GTH and the other reference methods on a data set comprising 300 64-molecule structures of bulk liquid water from *NVT* and *NpT* trajectories. As could perhaps be expected, the deviations of revPBE0-D3/def2-QZVP/AE, the same functional with tighter settings, are the smallest. On the other hand, ωB97X-rV/def2-QZVP/AE has considerably higher deviations, despite giving quite similar results to revPBE0-D3/def2-QZVP/AE. The distribution for MP2/cc-TZ/GTH is only slightly wider, even though it produces physical observables drastically different from those of the DFT methods. Notably, comparing these reference calculations with MLP predictions, the distribution of deviations for C-NNP@revPBE0-D3/TZV2P/GTH is similar in shape and magnitude (marginally wider, in fact) to that for revPBE0-D3/def2-QZVP/AE. This is despite the fact that in tests comparing actual performance in structure generation using MD, the C-NNP is difficult to distinguish from its reference method.^62^ This further questions the significance of reporting model errors. The cause of the differences between model predictions and reference calculations is the epistemic uncertainty of the MLP, which does not affect statistical physical observables in the same way as systematic differences between electronic structure methods, even if the deviations are of a comparable magnitude.

**Figure 7:**
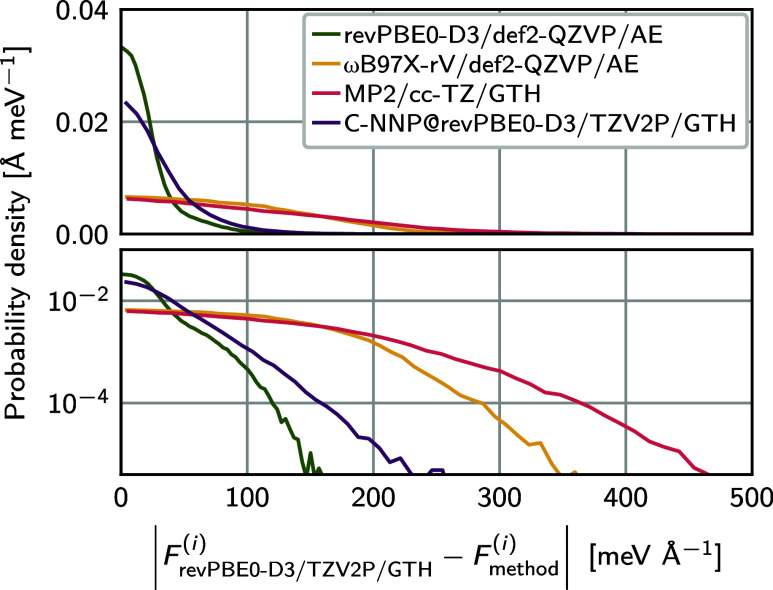
Distributions of absolute differences in the force components *F* ^(*i*)^ between revPBE0-D3/TZV2P/GTH and the three other DFT and post-HF methods, as well as the C-NNP trained to fit revPBE0-D3/TZV2P/GTH. The top panel shows the data on a linear scale, while the bottom panel uses a logarithmic scale on the *y*-axis to highlight the tails of the distributions. The comparison was done on a test set comprising 300 64-molecule bulk liquid water configurations.

## 4 Conclusions

In this work, we trained four C-NNPs to different reference electronic structure methods using an established training data set of aqueous systems that we extended to improve the density prediction and enable constant pressure simulations. After verifying that the transfer of the QbC-generated training data set to different reference methods does not degrade the models’ performance, we used the MLPs to calculate static and dynamic properties of water. The main focus was to compare two setups for revPBE0-D3, one that used well-established settings with a triple-ζ basis set and pseudopotentials, and one that used a quadruple-ζ basis set with an all-electron potential and tighter SCF convergence settings. The other two setups were the range-separated hybrid functional ωB97X-rV with the same highly converged settings, and MP2 using a moderately sized triple-ζ basis set and pseudopotentials.

When evaluating the models on an independent test set, we found that inaccuracies stemming from insufficient convergence in electronic structure calculations can lead to an increase in test set errors and we showed how using the GAPW method instead of GPW reduces these issues considerably. In comparisons of the physical observables calculated from MD trajectories, we consistently obtained considerably different results for revPBE0-D3/TZV2P/GTH and revPBE0-D3/def2-QZVP/AE and found that the switch to a larger basis set was more consequential than the switch to an all-electron potential. With the exception of the pressure–density curve, bringing the basis set closer to the convergence limit meant moving further away from experimental findings. With a quadruple-ζ basis set and all-electron potentials, ωB97X-rV emerged as the superior functional, although it was not able to match the experiments as closely as revPBE0-D3/TZV2P/GTH. Moreover, we confirmed that the use of MP2/cc-TZ/GTH with a triple-ζ basis set results in a severe overestimation of the hydrogen bonding strength, leading to highly structured water with insufficient diffusion.

Our results challenge the prevailing notion that triple-ζ basis sets and pseudopotentials are sufficient to accurately reveal the true performance of a given exchange–correlation functional for static and dynamic properties of aqueous systems. Instead, our results suggest that the impressive agreement of revPBE0-D3/TZV2P/GTH with the experimental data is not due to revPBE0-D3 being inherently excellent for water simulations, but rather due to some fortuitous cancellation of the errors of the functional itself and its numerical realization. This gives an additional perspective on previously identified cancellations within the revPBE0 functional viewed through the lens of the many-body expansion.^5^ Importantly, we offer a practical solution in the form of MLPs trained to highly converged electronic structure calculations that only need a modest number of expensive single-point calculations. This is enabled by uncertainty quantification using committee models, active learning, and the transferable nature of the resulting training sets. This approach allows one to easily consider multiple methods with converged settings and select the one that offers good performance for the studied system.

In comparison with our MP2 results, the fragment-based approaches by Willow et al.^57^ and Liu et al.^25^ show a substantially better match to experiment. This difference must be due to the differences in basis set, pseudopotential, or fragmentation, though the specifics were not investigated here or elsewhere. It is not clear from these results what the performance of fully converged MP2 would be for liquid water. The transfer learning strategy used by Chen et al.,^26^ which required only a moderate number of energies of 12-molecule periodic water structures to train an MLP at the level of correlated electronic structure methods (AFQMC and CCSD­(T)) yields a good match to experimental data. Consistent with the MB-pol model,^114,115^ their results indicate that an accurate description of aqueous systems can be obtained based on the CCSD­(T) level of theory. One of the cited approaches, our work, or some combination thereof could offer a pathway to accurate models based on correlated electronic structure close to the basis set limit for liquid water and more complex condensed-phase aqueous systems.

Overall, our results highlight both the importance of precise ab initio calculations and the role MLPs can play in enabling fully converged MD simulations based on popular electronic structure methods.

## Supplementary Material



## Data Availability

The input scripts, training data sets, and models underlying this study are openly available on Zenodo at 10.5281/zenodo.20718510.

## Notes

Additional details on construction of training data set and validation of C-NNPs; insights into the role of the pseudopotential; additional RDFs; further discussion about convergence settings and their role in model training (PDF)

## References

[1] Cisneros G. A., Wikfeldt K. T., Ojamäe L., Lu J., Xu Y., Torabifard H., Bartók A. P., Csányi G., Molinero V., Paesani F. (2016). Modeling molecular interactions in
water: From pairwise to many-body potential energy functions. Chem. Rev..

[2] Ceriotti M., Fang W., Kusalik P. G., McKenzie R. H., Michaelides A., Morales M. A., Markland T. E. (2016). Nuclear quantum
effects in water
and aqueous systems: Experiment, theory, and current challenges. Chem. Rev..

[3] Gillan M. J., Alfè D., Michaelides A. (2016). Perspective:
How good is DFT for
water?. J. Chem. Phys..

[4] Clark G. N.
I., Cappa C. D., Smith J. D., Saykally R. J., Head-Gordon T. (2010). The structure
of ambient water. Mol. Phys..

[5] Riera M., Lambros E., Nguyen T. T., Götz A. W., Paesani F. (2019). Low-order many- body interactions
determine the local
structure of liquid water. Chem. Sci..

[6] Gallo P., Amann-Winkel K., Angell C. A., Anisimov M. A., Caupin F., Chakravarty C., Lascaris E., Loerting T., Panagiotopoulos A. Z., Russo J., Sellberg J. A., Stanley H. E., Tanaka H., Vega C., Xu L., Pettersson L. G. M. (2016). Water:
A tale of two liquids. Chem. Rev..

[7] Bellissent-Funel M.-C., Hassanali A., Havenith M., Henchman R., Pohl P., Sterpone F., van der Spoel D., Xu Y., Garcia A. E. (2016). Water determines
the structure and dynamics of proteins. Chem.
Rev..

[8] Head-Gordon T., Johnson M. E. (2006). Tetrahedral structure or chains for liquid water. Proc. Natl. Acad. Sci. U.S.A..

[9] Liu J., He X., Zhang J. Z. H. (2017). Structure
of liquid water — a dynamical mixture
of tetrahedral and ‘ring-and-chain’ like structures. Phys. Chem. Chem. Phys..

[10] Liu J., He X., Zhang J. Z. H., Qi L.-W. (2018). Hydrogen-bond structure dynamics
in bulk water: insights from ab initio simulations with coupled cluster
theory. Chem. Sci..

[11] Wilkins D. M., Manolopoulos D. E., Pipolo S., Laage D., Hynes J. T. (2017). Nuclear
quantum effects in water reorientation and hydrogen-bond dynamics. J. Phys. Chem. Lett..

[12] Morawietz T., Singraber A., Dellago C., Behler J. (2016). How van der
Waals interactions
determine the unique properties of water. Proc.
Natl. Acad. Sci. U.S.A..

[13] de
Hijes P. M., Dellago C., Jinnouchi R., Kresse G. (2024). Density isobar of water and melting temperature of
ice: Assessing common density functionals. J.
Chem. Phys..

[14] Willow S. Y., Zeng X. C., Xantheas S. S., Kim K. S., Hirata S. (2016). Why is MP2-Water
‘cooler’ and ‘denser’ than DFT-Water?. J. Phys. Chem. Lett..

[15] Pestana L. R., Mardirossian N., Head-Gordon M., Head-Gordon T. (2017). Ab initio
molecular dynamics simulations of liquid water using high quality
meta-GGA functionals. Chem. Sci..

[16] Yao Y., Kanai Y. (2018). Free energy profile of NaCl in water: First-principles molecular
dynamics with SCAN and ωB97X-V exchange–correlation functionals. J. Chem. Theory Comput..

[17] Cheng B., Engel E. A., Behler J., Dellago C., Ceriotti M. (2019). Ab initio
thermodynamics of liquid and solid water. Proc.
Natl. Acad. Sci. U.S.A..

[18] Li M., Lan J., Wilkins D. M., Rybkin V. V., Iannuzzi M., Hutter J. (2025). Quantum dynamics
of water from Møller–Plesset perturbation theory via a
neural network potential. Commun. Comput. Chem..

[19] Ceriotti M., Cuny J., Parrinello M., Manolopoulos D. E. (2013). Nuclear
quantum effects and hydrogen bond fluctuations in water. Proc. Natl. Acad. Sci. U.S.A..

[20] Galib M., Duignan T. T., Misteli Y., Baer M. D., Schenter G. K., Hutter J., Mundy C. J. (2017). Mass density
fluctuations in quantum
and classical descriptions of liquid water. J. Chem. Phys..

[21] Villard J., Bircher M. P., Rothlisberger U. (2024). Structure
and dynamics of liquid
water from ab initio simulations: adding Minnesota density functionals
to Jacob’s ladder. Chem. Sci..

[22] Pestana L. R., Marsalek O., Markland T. E., Head-Gordon T. (2018). The quest
for accurate liquid water properties from first principles. J. Phys. Chem. Lett..

[23] Behler J., Parrinello M. (2007). Generalized neural-network representation of high-
dimensional potential-energy surfaces. Phys.
Rev. Lett..

[24] Martin-Barrios R., Navas-Conyedo E., Zhang X., Chen Y., Gulín-González J. (2024). An overview
about neural networks potentials in molecular dynamics simulation. Int. J. Quantum Chem..

[25] Liu J., Lan J., He X. (2022). Toward high-level
machine learning potential for water
based on quantum fragmentation and neural networks. J. Phys. Chem. A.

[26] Chen M. S., Lee J., Ye H.-Z., Berkelbach T. C., Reichman D. R., Markland T. E. (2023). Data-efficient
machine learning potentials from transfer learning of periodic correlated
electronic structure methods: Liquid water at AFQMC, CCSD, and CCSD­(T)
accuracy. J. Chem. Theory Comput..

[27] Dáo N. H., King D. S., Stein F., Wang X., Kim D., Brandenburg G., Hutter J., Cheng B. (2026). Systematic trends in
water properties across Jacob’s ladder density functionals. ChemRxiv.

[28] Schran C., Thiemann F. L., Rowe P., Müller E. A., Marsalek O., Michaelides A. (2021). Machine learning potentials for complex
aqueous systems made simple. Proc. Natl. Acad.
Sci. U.S.A..

[29] Omranpour A., Hijes P. M. D., Behler J., Dellago C. (2024). Perspective: Atomistic
simulations of water and aqueous systems with machine learning potentials. J. Chem. Phys..

[30] Advincula X. R., Schran C., Michaelides A. (2026). When is nanoconfined
water different
from interfacial water?. Faraday Discuss..

[31] Schmidt J., VandeVondele J., Kuo I.-F. W., Sebastiani D., Siepmann J. I., Hutter J., Mundy C. J. (2009). Isobaric-isothermal
molecular dynamics simulations utilizing density functional theory:
an assessment of the structure and density of water at near-ambient
conditions. J. Phys. Chem. B.

[32] Grimme S. (2011). Density functional
theory with London dispersion corrections. WIREs
Comput. Mol. Sci..

[33] Klimeš J., Michaelides A. (2012). Perspective: Advances and challenges in treating van
der waals dispersion forces in density functional theory. J. Chem. Phys..

[34] Dion M., Rydberg H., Schröder E., Langreth D. C., Lundqvist B. I. (2004). Van der
Waals density functional for general geometries. Phys. Rev. Lett..

[35] Vydrov O. A., Voorhis T. V. (2010). Nonlocal van der waals density functional: The simpler
the better. J. Chem. Phys..

[36] Yu Q., Qu C., Houston P. L., Nandi A., Pandey P., Conte R., Bowman J. M. (2023). A status
report on ‘gold standard’ machine-learned
potentials for water. J. Phys. Chem. Lett..

[37] O’Neill N., Shi B. X., Baldwin W. J., Witt W. C., Csányi G., Gale J. D., Michaelides A., Schran C. (2025). Towards routine condensed
phase simulations with delta–learned coupled cluster accuracy:
Application to liquid water. J. Chem. Theory
Comput..

[38] Ben M. D., Schönherr M., Hutter J., VandeVondele J. (2013). Bulk liquid
water at ambient temperature and pressure from MP2 theory. J. Phys. Chem. Lett..

[39] Ben M. D., Hutter J., VandeVondele J. (2015). Probing the
structural and dynamical
properties of liquid water with models including non-local electron
correlation. J. Chem. Phys..

[40] Markland T.
E., Ceriotti M. (2018). Nuclear quantum
effects enter the mainstream. Nat. Rev. Chem..

[41] Paesani F., Zhang W., Case D. A., Cheatham T. E., Voth G. A. (2006). An accurate
and simple quantum model for liquid water. J.
Chem. Phys..

[42] Paesani F., Voth G. A. (2009). The properties of water: Insights from quantum simulations. J. Phys. Chem. B.

[43] Wang L., Ceriotti M., Markland T. E. (2014). Quantum fluctuations and isotope
effects in ab initio descriptions of water. J. Chem. Phys..

[44] Marsalek O., Markland T. E. (2017). Quantum dynamics
and spectroscopy of ab initio liquid
water: The interplay of nuclear and electronic quantum effects. J. Phys. Chem. Lett..

[45] Habershon S., Markland T. E., Manolopoulos D. E. (2009). Competing
quantum effects in the
dynamics of a flexible water model. J. Chem.
Phys..

[46] Li X.-Z., Walker B., Michaelides A. (2011). Quantum nature of the hydrogen bond. Proc. Natl. Acad. Sci. U.S.A..

[47] Reinhardt A., Cheng B. (2021). Quantum-mechanical
exploration of the phase diagram of water. Nat.
Commun..

[48] Sharma B., Tran V. A., Pongratz T., Galazzo L., Zhurko I., Bordignon E., Kast S. M., Neese F., Marx D. (2021). A joint venture
of ab initio molecular dynamics, coupled cluster electronic structure
methods, and liquid-state theory to compute accurate isotropic hyperfine
constants of nitroxide probes in water. J. Chem.
Theory Comput..

[49] Kapil V., Schran C., Zen A., Chen J., Pickard C. J., Michaelides A. (2022). The first-principles phase diagram
of monolayer nanoconfined
water. Nature.

[50] Shen H., Chen L., Li J., Wang G. (2025). Neural network-assisted
analysis of free O–H orientational distribution at the air–water
interface: Gaussian or exponential?. J. Phys.
Chem. B.

[51] Cunningham O. S., Wilkins D. M. (2026). Learning electronic polarization in molecular systems:
Vibrational spectroscopy of ethanol–water mixtures. J. Chem. Inf. Model.

[52] Mardirossian N., Head-Gordon M. (2014). ωB97X-V: A 10-parameter, range-separated
hybrid,
generalized gradient approximation density functional with nonlocal
correlation, designed by a survival-of-the-fittest strategy. Phys. Chem. Chem. Phys..

[53] Becke A. D. (1997). Density-functional
thermochemistry. V. systematic optimization of exchange-correlation
functionals. J. Chem. Phys..

[54] Mardirossian N., Head-Gordon M. (2014). Exploring
the limit of accuracy for density functionals
based on the generalized gradient approximation: Local, global hybrid,
and range- separated hybrid functionals with and without dispersion
corrections. J. Chem. Phys..

[55] Brauer B., Kesharwani M. K., Kozuch S., Martin J. M. L. (2016). The S66 ×
8 benchmark for noncovalent interactions revisited: explicitly correlated
ab initio methods and density functional theory. Phys. Chem. Chem. Phys..

[56] Manna D., Kesharwani M. K., Sylvetsky N., Martin J. M. L. (2017). Conventional
and explicitly correlated ab initio benchmark study on water clusters:
Revision of the BEGDB and WATER27 data sets. J. Chem. Theory Comput..

[57] Willow S. Y., Salim M. A., Kim K. S., Hirata S. (2015). Ab initio
molecular
dynamics of liquid water using embedded-fragment second-order many-body
perturbation theory towards its accurate property prediction. Sci. Rep..

[58] Lee H.-S., Tuckerman M. E. (2007). Dynamical
properties of liquid water from ab initio
molecular dynamics performed in the complete basis set limit. J. Chem. Phys..

[59] Wang J., Román-Pérez G., Soler J. M., Artacho E., Fernández-Serra M.-V. (2011). Density,
structure, and dynamics
of water: The effect of van der Waals interactions. J. Chem. Phys..

[60] Oneill N., Shi B. X., Baldwin W. J., Bartok A. P., Pickard C. J., Michaelides A., Csanyi G., Berkelbach T. C. (2026). How reproducible
are first-principles simulations of liquid water?. arXiv.

[61] Kuryla D., Berger F., Csányi G., Michaelides A. (2025). How accurate
are DFT forces? unexpectedly large uncertainties in molecular datasets. J. Chem. Phys..

[62] Schran C., Brezina K., Marsalek O. (2020). Committee
neural network potentials
con- trol generalization errors and enable active learning. J. Chem. Phys..

[63] Kühne T. D., Iannuzzi M., Ben M. D., Rybkin V. V., Seewald P., Stein F., Laino T., Khaliullin R. Z., Schütt O., Schiffmann F. (2020). CP2K: An electronic
structure and molecular dynamics software package - Quickstep: Efficient
and accurate electronic structure calculations. J. Chem. Phys..

[64] Vandevondele J., Krack M., Mohamed F., Parrinello M., Chassaing T., Hutter J. (2005). Quickstep: Fast and
accurate density
functional calculations using a mixed Gaussian and plane waves approach. Comput. Phys. Commun..

[65] Perdew J. P., Burke K., Ernzerhof M. (1996). Generalized
gradient approximation
made simple. Phys. Rev. Lett..

[66] Zhang Y., Yang W. (1998). Comment on ‘generalized
gradient approximation made simple’. Phys. Rev. Lett..

[67] Adamo C., Barone V. (1999). Toward reliable density
functional methods without
adjustable parameters: The PBE0 model. J. Chem.
Phys..

[68] Grimme S., Antony J., Ehrlich S., Krieg H. (2010). A consistent and accurate
ab initio parametrization of density functional dispersion correction
(DFT-D) for the 94 elements H-Pu. J. Chem. Phys..

[69] Goerigk L., Grimme S. (2011). A thorough benchmark of density functional methods
for general main group thermochemistry, kinetics, and noncovalent
interactions. Phys. Chem. Chem. Phys..

[70] Lippert G., Hutter J., Parrinello M. (1997). A hybrid Gaussian
and plane wave
density functional scheme. Mol. Phys..

[71] Goedecker S., Teter M., Hutter J. (1996). Separable
dual-space Gaussian pseudopotentials. Phys.
Rev. B.

[72] Krack M. (2005). Pseudopotentials
for H to Kr optimized for gradient-corrected exchange- correlation
functionals. Theor. Chem. Acc..

[73] Guidon M., Hutter J., VandeVondele J. (2009). Robust periodic
hartree-fock exchange
for large-scale simulations using Gaussian basis sets. J. Chem. Theory Comput..

[74] Guidon M., Hutter J., VandeVondele J. (2010). Auxiliary density matrix methods
for Hartree–Fock exchange calculations. J. Chem. Theory Comput..

[75] Lippert G., Hutter J., Parrinello M. (1999). The Gaussian and augmented-plane-wave
density functional method for ab initio molecular dynamics simulations. Theor. Chem. Acc..

[76] Krack M., Parrinello M. (2000). All-electron ab-initio molecular dynamics. Phys. Chem. Chem. Phys..

[77] Weigend F., Ahlrichs R. (2005). Balanced basis sets of split valence, triple zeta valence
and quadruple zeta valence quality for H to Rn: Design and assessment
of accuracy. Phys. Chem. Chem. Phys..

[78] Sabatini R., Gorni T., de Gironcoli S. (2013). Nonlocal van
der Waals density functional
made simple and efficient. Phys. Rev. B.

[79] Mardirossian N., Pestana L. R., Womack J. C., Skylaris C.-K., Head-Gordon T., Head-Gordon M. (2017). Use of the rVV10 nonlocal correlation
functional in
the B97M-V density functional: Defining B97M-rV and related functionals. J. Phys. Chem. Lett..

[80] Møller C., Plesset M. S. (1934). Note on an approximation
treatment for many-electron
systems. Phys. Rev..

[81] Ben M. D., Hutter J., VandeVondele J. (2012). Second-order
Møller–Plesset
perturbation theory in the condensed phase: An efficient and massively
parallel Gaussian and plane waves approach. J. Chem. Theory Comput..

[82] Ben M. D., Hutter J., VandeVondele J. (2013). Electron correlation
in the condensed
phase from a resolution of identity approach based on the Gaussian
and plane waves scheme. J. Chem. Theory Comput..

[83] Ben M. D., Hutter J., VandeVondele J. (2015). Forces and stress in second order
Møller– Plesset perturbation theory for condensed phase
systems within the resolution-of-identity Gaussian and plane waves
approach. J. Chem. Phys..

[84] Vahtras O., Almlöf J., Feyereisen M. W. (1993). Integral approximations for LCAO-SCF
calculations. Chem. Phys. Lett..

[85] Weigend F., Häser M., Patzelt H., Ahlrichs R. (1998). RI-MP2: optimized auxiliary
basis sets and demonstration of efficiency. Chem. Phys. Lett..

[86] Behler J. (2011). Atom-centered
symmetry functions for constructing high-dimensional neural network
potentials. J. Chem. Phys..

[87] Singraber A., Behler J., Dellago C. (2019). Library-based LAMMPS implementation
of high-dimensional neural network potentials. J. Chem. Theory Comput..

[88] Singraber A., Morawietz T., Behler J., Dellago C. (2019). Parallel multistream
training of high-dimensional neural network potentials. J. Chem. Theory Comput..

[89] Nguyen D. H., Widrow B. (1990). Improving the learning speed of 2-layer
neural networks
by choosing initial values of the adaptive weights. Proc. Int. Joint Conf. Neural Netw..

[90] Shah S., Palmieri F., Datum M. (1992). Optimal filtering algorithms
for
fast learning in feedforward neural networks. Neural Netw..

[91] Blank T. B., Brown S. D. (1994). Adaptive, global, extended kalman filters for training
feedforward neural networks. J. Chemom.

[92] Kapil V., Rossi M., Marsalek O., Petraglia R., Litman Y., Spura T., Cheng B., Cuzzocrea A., Meißner R. H., Wilkins D. M. (2019). i-PI
2.0: A universal
force engine for advanced molecular simulations. Comput. Phys. Commun..

[93] Bussi G., Donadio D., Parrinello M. (2007). Canonical
sampling through velocity
rescaling. J. Chem. Phys..

[94] Craig I. R., Manolopoulos D. E. (2004). Quantum
statistics and classical mechanics: Real time
correlation functions from ring polymer molecular dynamics. J. Chem. Phys..

[95] Rossi M., Ceriotti M., Manolopoulos D. E. (2014). How to
remove the spurious resonances
from ring polymer molecular dynamics. J. Chem.
Phys..

[96] Ceriotti M., Parrinello M., Markland T. E., Manolopoulos D. E. (2010). Efficient
stochastic thermostatting of path integral molecular dynamics. J. Chem. Phys..

[97] Bussi G., Zykova-Timan T., Parrinello M. (2009). Isothermal-isobaric molecular dynamics
using stochastic velocity rescaling. J. Chem.
Phys..

[98] Ceriotti M., Bussi G., Parrinello M. (2009). Langevin equation with colored noise
for constant-temperature molecular dynamics simulations. Phys. Rev. Lett..

[99] Ceriotti M., Bussi G., Parrinello M. (2010). Colored-noise
thermostats á
la carte. J. Chem. Theory Comput..

[100] Dünweg B., Kremer K. (1993). Molecular dynamics
simulation of
a polymer chain in solution. J. Chem. Phys..

[101] Luzar A., Chandler D. (1993). Structure and hydrogen
bond dynamics
of water–dimethyl sulfoxide mixtures by computer simulations. J. Chem. Phys..

[102] Luzar A., Chandler D. (1996). Hydrogen-bond kinetics in liquid
water. Nature.

[103] Rapaport D. C. (1983). Hydrogen bonds in water. Mol.
Phys..

[104] Durham B., Probert M. I. J., Hasnip P. J. (2025). Beating the egg-box
effect in plane-wave DFT simulations. Electron.
Struc..

[105] Skinner L. B., Huang C., Schlesinger D., Pettersson L. G. M., Nilsson A., Benmore C. J. (2013). Benchmark oxygen-oxygen
pair-distribution function of ambient water from x-ray diffraction
measurements with a wide Q-range. J. Chem. Phys.

[106] Ma Z., Zhang Y., Tuckerman M. E. (2012). Ab initio molecular dynamics study
of water at constant pressure using converged basis sets and empirical
dispersion corrections. J. Chem. Phys..

[107] de Hijes P. M., Dellago C., Jinnouchi R., Schmiedmayer B., Kresse G. (2024). Comparing machine learning potentials
for water: Kernel-based regression and Behler–Parrinello neural
networks. J. Chem. Phys..

[108] Grindley T., Lind J. E. (1971). PVT properties
of water and mercury. J. Chem. Phys..

[109] Rappoport D., Furche F. (2010). Property-optimized
Gaussian basis
sets for molecular re- sponse calculations. J. Chem. Phys..

[110] Afzal M. A. F., Hachmann J. (2019). Benchmarking DFT approaches
for the
calculation of polarizability inputs for refractive index predictions
in organic polymers. Phys. Chem. Chem. Phys..

[111] Kumar R., Luber S. (2025). Electric dipole polarizability
calculation
for periodic and non-periodic systems using atomic-orbitals-based
linear response theory, Helv. Chim. Acta.

[112] Sanz E., Vega C., Abascal J. L. F., MacDowell L. G. (2004). Phase diagram
of water from computer simulation. Phys. Rev.
Lett..

[113] Holz M., Heil S. R., Sacco A. (2000). Temperature-dependent
self-diffusion coefficients of water and six selected molecular liquids
for calibration in accurate 1H NMR PFG measurements. Phys. Chem. Chem. Phys..

[114] Medders G. R., Babin V., Paesani F. (2014). Development of a “first-principles”
water potential with flexible monomers. III. liquid phase properties. J. Chem. Theory Comput..

[115] Zhu X., Riera M., Bull-Vulpe E. F., Paesani F. (2023). MB-pol­(2023): Sub-chemical
accuracy for water simulations from the gas to the liquid phase. J. Chem. Theory Comput..

