# Supplementary material for: More Converged, Less Accurate? Reassessing Standard Choices for Ab Initio Water Using Machine Learning Potentials
Source: J Phys Chem B. 2026 Jul 6;130(28):7215–26. doi: 10.1021/acs.jpcb.6c02917 (PMC13383747; doi:10.1021/acs.jpcb.6c02917)
Supplement: Supplementary file 1 [file jp6c02917_si_001.pdf]

Supporting information for: More Converged, Less Accurate? Reassessing Standard Choices for Ab Initio Water Using Machine Learning Potentials

Hubert Beck and Ondrej Marsalek[a)]

Charles University, Faculty of Mathematics and Physics, Ke Karlovu 3, 121 16 Prague 2, Czech Republic

(Dated: 19 June 2026)

[a)] Electronic mail: ondrej.marsalek@matfyz.cuni.cz

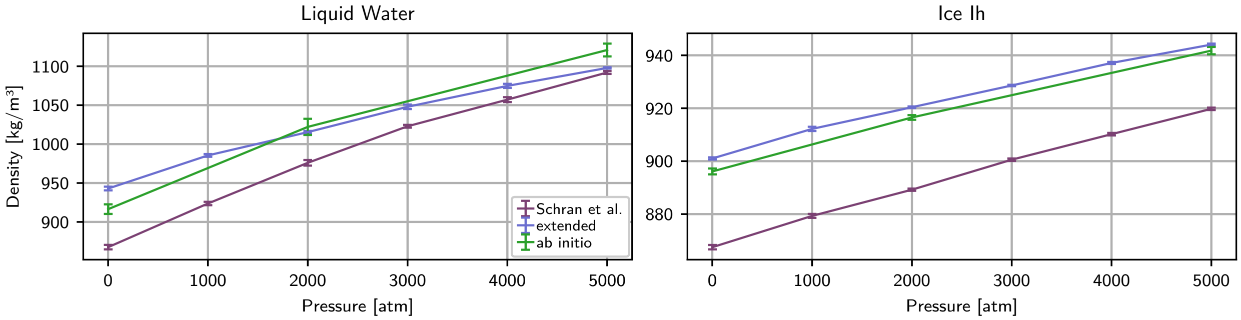

FIG. S1. Comparison of pressure–density curves from ab initio simulations and C-NNPs trained on two different training sets. The setup for the ab initio simulations and the two training sets is revPBE0-D3/TZV2P/GTH. Schran et al. corresponds to the dataset originally published in reference S1, "extended" to the version that we extended by 150 NpT structures. The left panel displays the pressure–density curve for bulk liquid water, the right panel for ice I_h. Error bars were obtained using block averaging.

S1. ADAPTING THE TRAINING DATASET

The water training dataset published by Schran et al.[S1] consists of 814 structures of bulk liquid water, ice, and a slab with a liquid–vacuum interface sampled from the canonical ensemble (NVT). Committee neural network potentials (C-NNPs) trained on this dataset are able to reproduce many physical observables from ab initio simulations with the same reference method. However, as Figure S1 shows, the models underestimate the density of both liquid water and ice I_h. Therefore, we extended the training dataset by adding structures from the isobaric–isothermal ensemble (NpT) simulated with the C-NNP at pressures of 1, 2000 and 5000 atm using a query by committee (QbC) workflow.[S1] In total, we added 110 64-molecule structures of liquid water and 40 92-molecule structures of ice I_h. Although these configurations were obtained from simulations using a model that produces the wrong density, including them into the training set with the correct energies and forces from ab initio calculations considerably improves the density predictions. For liquid water (shown in the left panel of Figure S1), the density does not match the results from ab initio simulations perfectly, but this is likely due to insufficient convergence of the expensive ab initio trajectories.

	wall time	memory	resources
revPBE0-D3/TZV2P/GTH	0:04	18 GB	1 EPYC 7301 node
revPBE0-D3/def2-QZVP/AE	0:14	35 GB	1 EPYC 7301 nodes
ωB97X-rV/def2-QZVP/AE	0:34	123 GB	1 EPYC 7301 node
MP2/cc-TZ/GTH	0:28	3.2 TB	16 EPYC 7H12 nodes

TABLE I. Computational costs for a single point calculation of a 64-molecule structure of bulk liquid water.

	wall time	memory	resources
revPBE0-D3/TZV2P/GTH	0:05	24 GB	1 EPYC 7301 node
revPBE0-D3/def2-QZVP/AE	0:20	55 GB	1 EPYC 7301 node
ωB97X-rV/def2-QZVP/AE	0:54	185 GB	1 EPYC 7301 node
MP2/cc-TZ/GTH	4:20	3.1 TB	16 EPYC 7H12 nodes

TABLE II. Computational costs for a single point calculation of a 96-molecule structure of ice I_h.

	wall time	memory	resources
revPBE0-D3/TZV2P/GTH	0:11	54 GB	1 EPYC 7301 node
revPBE0-D3/def2-QZVP/AE	0:28	760 GB	2 EPYC 7H12 nodes
ωB97X-rV/def2-QZVP/AE	0:35	952 GB	4 EPYC 7H12 nodes

TABLE III. Computational costs for a single point calculation of a 216-molecule slab of liquid water. Note the switch to larger nodes compared to liquid water and ice calculations for the highly converged setups. One EPYC 7031 node contains two AMD EPYC 7031 16-core CPUs. One EPYC 7H12 node contains two AMD EPYC 7H12 64-core CPUs. The wall time is given in the format hours:minutes.

S2. RESOURCE REQUIREMENTS

Tables I, II, and III show the computational costs for a single point calculation for the different methods for one configuration of liquid water, ice I_h, and slab, respectively. In each calculation, the SCF was initialized with converged Kohn–Sham orbitals from corresponding GGA calculations to speed up convergence. Please note that an important reason for the increased demands in CPU time and memory of ωB97X-rV/def2-QZVP/AE compared to revPBE0-D3/def2-QZVP/AE is that ωB97X-rV/def2-QZVP/AE requires a lower Schwarz

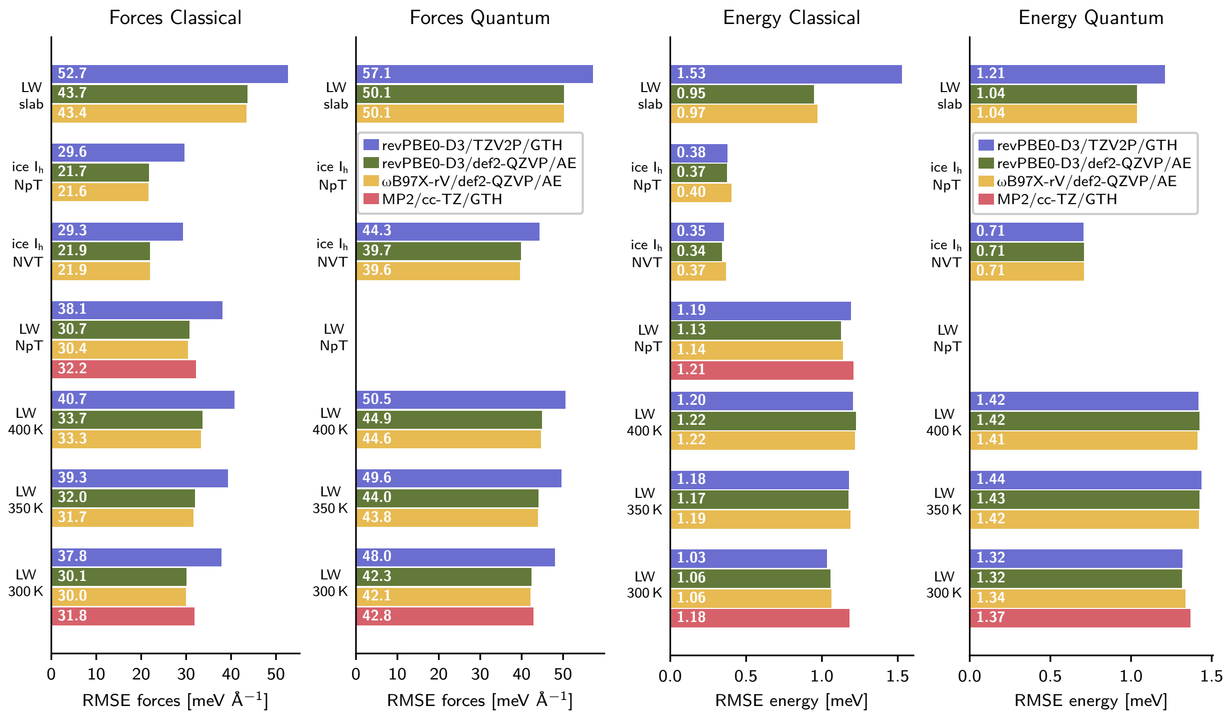

FIG. S2. Test set errors of the four main models. The two panels on the left display the force RMSEs for classical and quantum structures and complete Figure 1 of the main paper. The two panels on the right display the energy RMSEs for the same test sets.

inequality threshold, which controls the screening of near-field electronic repulsion integrals. The lower threshold results in a substantially higher number in integrals during the SCF procedure. Furthermore, during the calculation of the correlation energy for MP2, an MPI group size of one process was used for liquid water, and two processes for ice I_h. This change results in a lower memory requirement but longer wall time for ice I_h calculations.

S3. COMPLETE TEST SETS

Figure S2 shows the test set errors of the four main models for both energy and forces. The two panels on the left display the force errors and complete Figure 1 of the main article. The additional test sets are fully consistent with the observations made in the main paper. The energy RMSEs displayed in the right panels show that for most test sets the energy errors are very consistent. The increase in test errors for the revPBE0-D3/TZV2P/GTH datasets due to the egg box effect, which we observed in the force errors, is absent in most

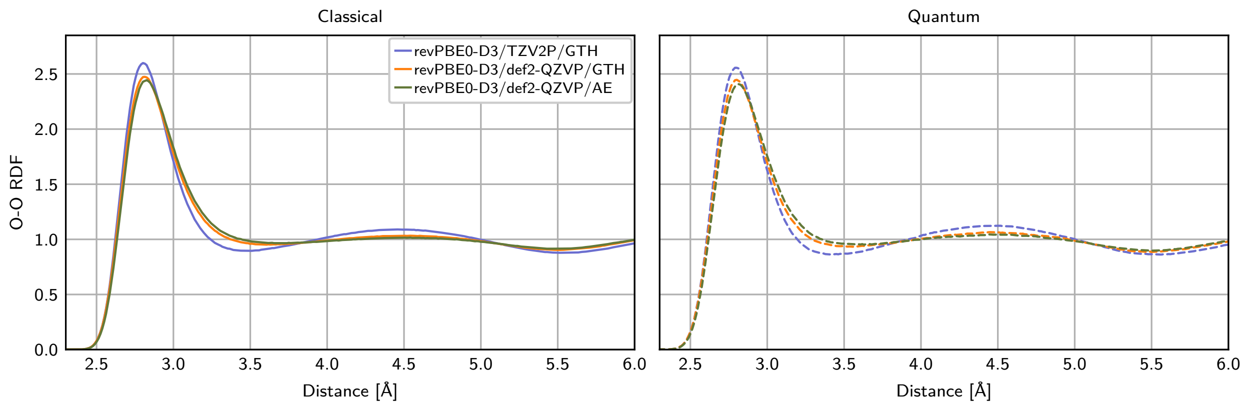

FIG. S3. The oxygen-oxygen RDF for the 3 revPBE0-D3 C-NNPs. The results from the classical MD simulations are shown in the left panel, the PIMD results are shown in the right panel.

test sets. The only exception is the slab test sets, where revPBE0-D3/TZV2P/GTH has a considerably higher error. The reason why the slab structures are an exception and why the energy error is higher for classical slab configurations than for quantum slab configurations remains unclear.

S4. ROLE OF BASIS SETS AND PSEUDOPOTENTIALS

In the main paper, we compared a standard setup for revPBE0-D3 with one that was highly converged. The main differences between the two setups were the more extensive basis set and the use of an all-electron potential instead of GTH pseudopotentials[S2] for the highly converged setup. More details can be found in the computational details (Section II) of the main article. In this section, we will show in greater detail the individual contributions of the basis set and potential by presenting the results for a model trained to fit the revPBE0-D3/def2-QZVP/GTH setup and comparing them with the results of the original revPBE0-D3/TZV2P/GTH model and the fully converged revPBE0-D3/def2-QZVP/AE model already shown in the main paper.

Radial Distribution Function

Figure S3 shows the oxygen–oxygen radial distribution function (RDF) for classical and quantum simulations. The RDFs of the revPBE0-D3/def2-QZVP/GTH model are very

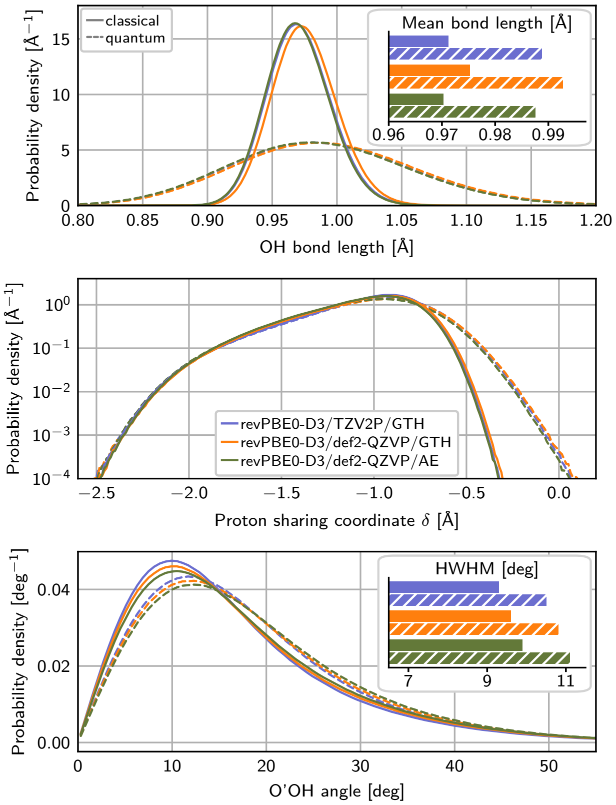

FIG. S4. Analysis of the structure of covalent and hydrogen bonds. Top panel: The distribution and means of lengths of covalent oxygen-hydrogen bonds for classical (solid lines/bars) and PI (dashed lines/bars) MD run with the 3 different C-NNPs. The main plot shows the distributions of bond lengths and the inset the mean bond lengths. Middle panel: The distributions of the proton sharing coordinate. Bottom panel: The distributions of the hydrogen bond angle. The inset compares the half width at half maximum of the different methods.

similar to those of the revPBE0-D3/def2-QZVP/AE model, while there are considerable differences from the revPBE0-D3/TZV2P/GTH RDFs. This indicates that for distances around the first coordination shell and beyond, the pseudopotentials are a good approximation to the physically accurate all-electron potentials. On the other hand, changing the basis set has a considerable effect on the RDF, which is discussed in greater detail in the main article.

Covalent and Hydrogen Bonds

Figure S4 shows an analysis of the covalent and hydrogen bonds of the three revPBE0-D3 models, analogous to Figure 3 in the main article. The distribution of bond lengths shown in the top panel shows only minuscule differences between revPBE0-D3/TZV2P/GTH and revPBE0-D3/def2-QZVP/AE. While it can be expected that revPBE0-D3/def2-QZVP/GTH, which is a mix of these two setups, gives a similar distribution, we see a clear shift towards longer covalent bonds, especially for the classical system. We conclude that in our findings the improved basis set increases the bond lengths, whereas the all-electron potential has the opposite effect, leading to a high similarity between the state-of-the-art and the fully converged setup. This indicates that in close proximity to the nuclei, there is a measurable but not disastrous difference between pseudopotentials and all-electron potentials. In the hydrogen bonds, we see less consequential differences between the models. The δ-coordinate plotted in the center panel of Figure S4 displays only small differences between the three models. As with the covalent bonds, the δ-coordinate distribution of revPBE0-D3/def2-QZVP/GTH is shifted slightly to the right compared to the other two, suggesting that the changes in basis set and potential compensate each other. The distribution of the hydrogen bond angles in the bottom panel shows that the distribution of revPBE0-D3/def2-QZVP/GTH is in the middle of the other two models, illustrating that both the increased basis set and the upgraded potential impact the angular distribution of the hydrogen bond in the same direction.

Pressure–Density Curve

Figure S5 shows the pressure–density curve for the three revPBE0-D3 models. For liquid water, which is shown in the left panel, revPBE0-D3/def2-QZVP/GTH and revPBE0-D3/def2-QZVP/AE are in almost perfect agreement with each other and the experimental reference curve.[S3] Their density is consistently around $50\,\mathrm{kg\,m^{-3}}$ higher than the revPBE0-D3/TZV2P/GTH density. The differences between classical and quantum simulations are small for all models. In the right panel of Figure S5 the pressure–density curves for ice $\mathrm{I_h}$ are plotted and display a different behavior than the bulk liquid water curves. Here, the density corresponding to the revPBE0-D3/def2-QZVP/GTH models is slightly higher than the

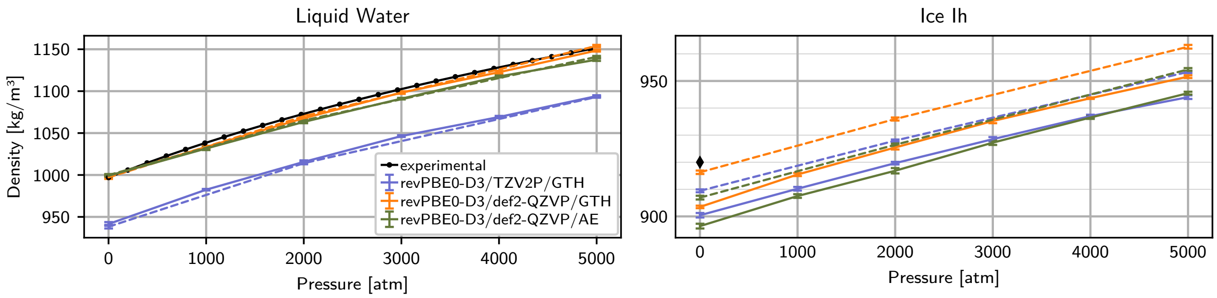

FIG. S5. The pressure–density curve of liquid water (left) and ice I_h(right) for the three different C-NNPs. The black curve in the left panel and the black diamond in the right panel shows the experimental reference[S3,S4] for each system. The error bars indicate statistical errors obtained by block averaging. As in the other plots, solid lines are used for classical MD and dashed lines for PIMD.

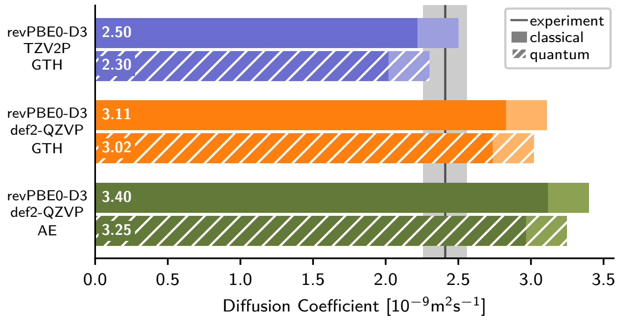

FIG. S6. Comparison of the diffusion coefficients for the different C-NNPs in classical and quantum MD. The pale colors at the end of each bar signals the magnitude of the finite size corrections added to the calculated values. The gray vertical area indicates the experimental reference value.[S5]

other two revPBE0-D3 models, with discrepancies of less than $10\,\mathrm{kg\,m^{-3}}$.

Diffusion Coefficient

Figure S6 shows the diffusion coefficients for the three revPBE0-D3 models, analogous to Figure 5 in the main paper. The diffusion coefficient for revPBE0-D3/def2-QZVP/GTH lies in between the two other revPBE0-D3 models, closer to the highly converged setup. This indicates that while both changes have a meaningful impact, the increased basis set has a stronger effect than the change in potential. For all three models, we observe a slightly

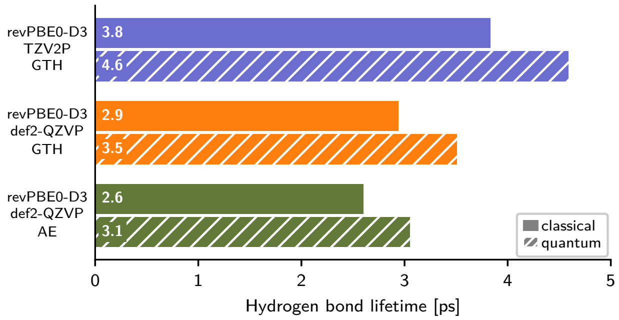

FIG. S7. Hydrogen bond lifetimes for the 3 different revPBE0-D3 C-NNPs.

decreased diffusion coefficient when including nuclear quantum effects.

Hydrogen Bond Lifetimes

Figure S7 shows the hydrogen bond lifetimes for the three revPBE0-D3 models, analogous to Figure 6 in the main paper. As with the diffusion constant, the results of revPBE0-D3/def2-QZVP/GTH lie in between the results of the other two models, with a stronger similarity to the revPBE0-D3/def2-QZVP/AE setup. This further highlights the strong connection between hydrogen bond lifetimes and diffusion.

Conclusion

We conclude that the results of revPBE0-D3/def2-QZVP/GTH lie between those of revPBE0-D3/TZV2P/GTH and revPBE0-D3/def2-QZVP/AE for most of the physical observables that we investigated. This indicates that the increased size of the basis set and the all-electron potential impact the water systems in similar ways. The notable exception were the distributions of covalent bond lengths and the proton sharing coordinate, where the two changes cancel each other out almost perfectly. For observables where intermolecular interactions play a considerable role, the effects of increasing the size of the basis set were somewhat stronger than those of switching to an all-electron potential. While these results highlight the high quality of the GTH pseudopotentials available in CP2K, we should note this is a generic parametrization for PBE. A parametrization that more closely fits our converged setup could further decrease the error relative to the all-electron model.

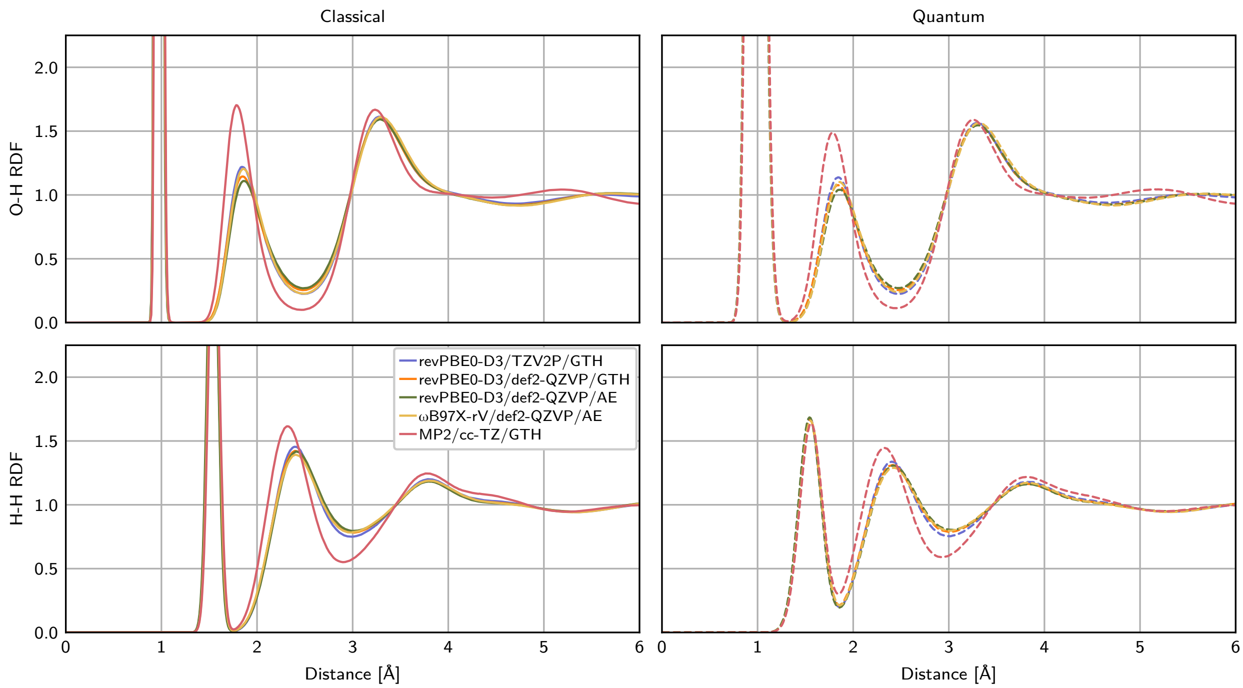

FIG. S8. The oxygen–hydrogen (top panel) and hydrogen–hydrogen (bottom) RDF for the five C-NNPs discussed in the main article and here. The results from the classical MD simulations are shown in the left panels, the PIMD results are shown in the right panels.

S5. OTHER RADIAL DISTRIBUTION FUNCTIONS

Figure S8 shows the oxygen-hydrogen (top panels) and hydrogen-hydrogen (bottom panels) RDFs for the four models from the main paper, as well as the revPBE0-D3/def2-QZVP/GTH model. The general trends, which we have already observed for the oxygen-oxygen RDF in Figure 2 of the main article, are confirmed here as well. In particular, the RDFs for the MP2/cc-TZ/GTH model are notably more structured than the RDFs of the other models, with peaks and valleys occurring at a shorter distance. Even for longer distances, considerable differences in the structure can still be observed. When comparing revPBE0-D3/TZV2P/GTH and revPBE0-D3/def2-QZVP/AE, the trend of a smoother RDF for the highly converged setup is confirmed, especially for the first inter-molecular peak of the O-H RDF. However, the trend that the ωB97X-rV/def2-QZVP/AE and revPBE0-D3/def2-QZVP/AE models display a similar RDF as observed for the O-O RDF is broken. Now, the O-H RDF of ωB97X-rV/def2-QZVP/AE shows greater similarity with revPBE0-D3/TZV2P/GTH.

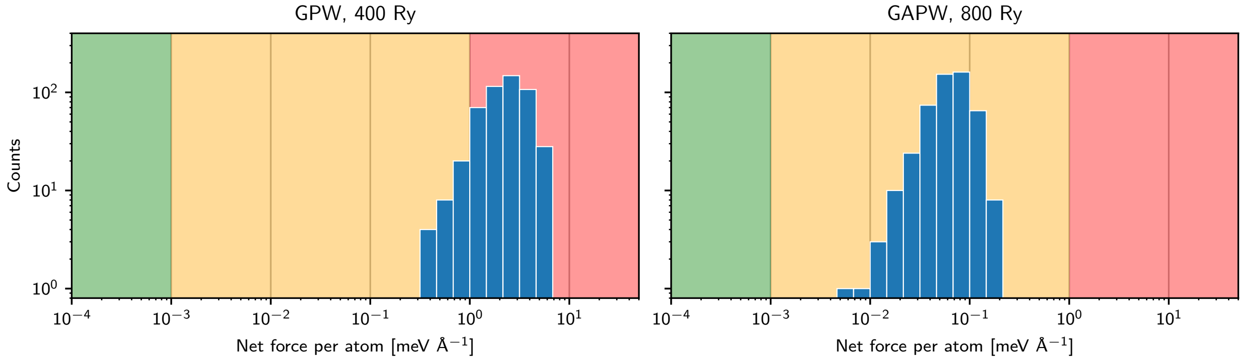

FIG. S9. The distribution of net forces per structure for the classical liquid water NpT test set. In the left panel the standard revPBE0-D3/TZV2P/GTH setup (with GPW and a 400 Ry plane wave cutoff) as used in the main paper is shown, in the right panel instead the GAPW method with a 800 Ry cutoff was employed. The background colors correspond to the thresholds used in reference S6. The red color above 1 meV Å^{-1} indicates problematic errors, the orange color between 10^{-3} and 1 meV Å^{-1} corresponds to acceptable errors and the green area below 10^{-3} meV Å^{-1} means negligible errors.

S6. UNDER-CONVERGED DATASETS

In this section, we will further investigate under-converged datasets and their effect on model testing and training. Under-converged in this case does not refer to the type of Gaussian basis sets or potentials, but to convergence settings, plane wave cutoffs and calculation methods. We will look into the net forces of systems, the strength of the sum over all forces in the system, which should be 0. They have been shown to be a useful tool for finding unreliable electronic structure calculations.[S6] One of the primary causes for errors in calculations using plane wave basis sets is the so called "egg box effect".[S7] The egg box effect results in different total energies of identical systems based on their location relative to the real space computational grid of the plane wave basis set. In case of a low plane wave energy cutoff, this grid is coarse, resulting in inconsistencies.

Net Forces

Figure S9 shows the distribution of net forces per atom for two versions of the classical liquid water NpT test set, which consists of 500 structures and was one of the test sets used

for Figure 1 of the main paper. The area with the red-shaded background color indicates a magnitude of net forces that is indicative of problems in the calculation setup. The left panel shows the net forces when calculating the test set with the standard revPBE0-D3/ TZV2P/GTH setup, and a large part of the distribution is in the red zone. The right panel of Figure S9 shows the result for the test set with the same basic DFT setup, but instead of the Gaussian and plane waves (GPW) method,[S8] it used the Gaussian and augmented plane waves (GAPW) method[S9] in addition to an increased plane wave cutoff. This change in the setup considerably improves the net forces by decreasing them by more than one order of magnitude. For the 64-atom structures, this means a reduction in the total net forces per structure from 486 meV Å^{-1} to 13 meV Å^{-1}. This is lower than the 40 meV Å^{-1} that Kuryla et al.[S6] achieved for a similar water setup in CP2K with a plane wave cutoff of 1200 Ry. It shows that the GAPW method is an important tool in improving the quality of datasets calculated in CP2K. This improvement in the quality of the test data set leads to an immediate improvement in the test score. When testing the revPBE0-D3/TZV2P/GTH model on the original dataset, we obtain a force RMSE of 38.1 meV Å^{-1}. Using the same model on the improved test set, we get a force RMSE of 33.8 meV Å^{-1}, despite technically using a different reference method from the training data.

Figure S10 shows the distribution of net forces per atom for the collective test sets for all four electronic structure calculation setups. The dataset is the full test set established by Schran et al.[S1] extended by a set of 500 structures from NpT simulations of bulk water, which was already used in Figure S9. The two top panels display the distribution of the two revPBE0-D3 datasets, further proving that the GAPW method reduces inconsistencies in DFT calculations. The distributions of revPBE0-D3/def2-QZVP/AE (top right) and ωB97X-rV/def2-QZVP/AE (bottom left) are very similar, highlighting that the improvement is largely independent from the exchange–correlation functional. The distribution of the MP2/cc-TZ/GTH setup (bottom right) is shifted further to the left by almost two orders of magnitude, with almost half of the structures scoring below the 10^{-3} meV Å^{-1} threshold. The MP2 distribution is smaller because only a subset of the full test set could be calculated due to the expensive nature of bulk MP2 calculations.

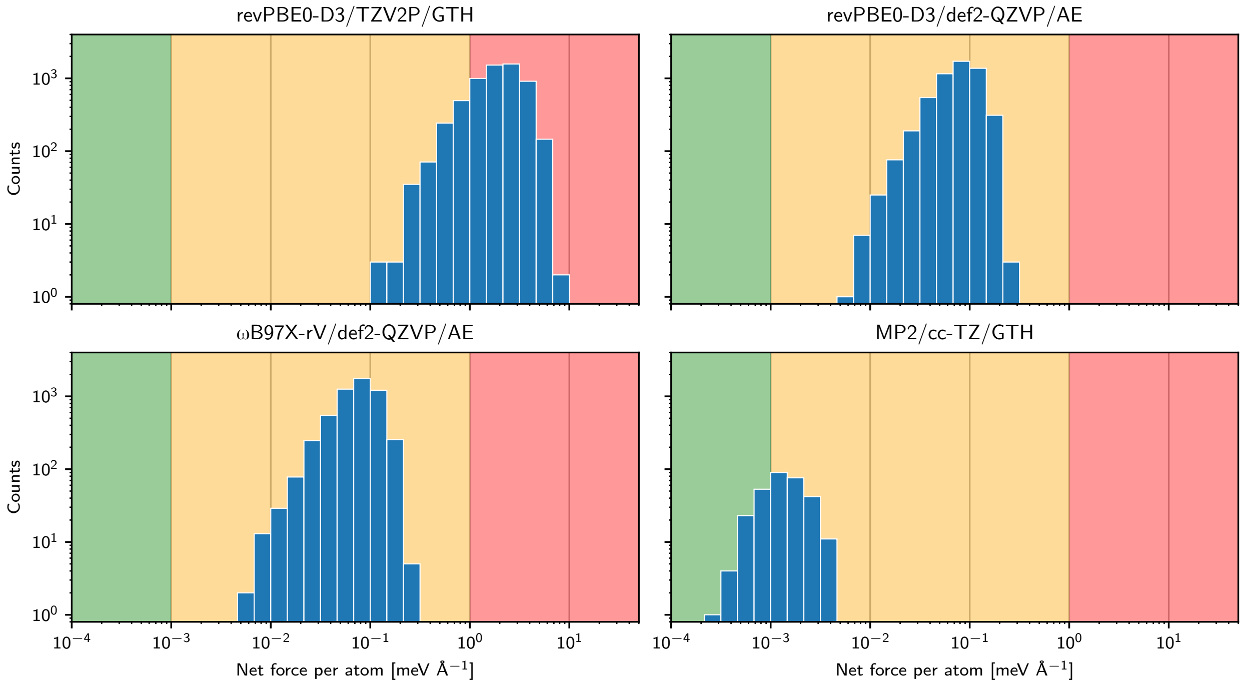

FIG. S10. The distribution of net forces per structure for the full test sets for the four main reference methods. The background colors correspond to the thresholds used in reference S6. The red color above 1 meV Å^{-1} indicates problematic errors, the orange color between 10^{-3} and 1 meV Å^{-1} corresponds to acceptable errors and the green area below 10^{-3} meV Å^{-1} means negligible errors.

Effect of the Egg Box Effect on Training

We have highlighted how the egg box effect is a primary source of error in calculations using plane wave basis set and how these errors can be diminished by the GAPW method. Furthermore, we showed how these errors lead to an overestimation in a model's test error as the evaluation of a single structure is more strongly affected by these problems than the training of a model with a large set of structures. Here, we investigate further how this error affects the training by comparing models with different architectures and capacities on the same training datasets. In addition to the N2P2[S10,S11] models used in the main article, NequIP[S12] and MACE[S13] models were trained using the revPBE0-D3/TZV2P/GTH and revPBE0-D3/def2-QZVP/AE training sets. For NequIP, we trained one model with commonly used settings, which amounts to a total of 154 000 trainable parameters and one extremely minimalist model containing just 11 000 parameters. The MACE model uses some

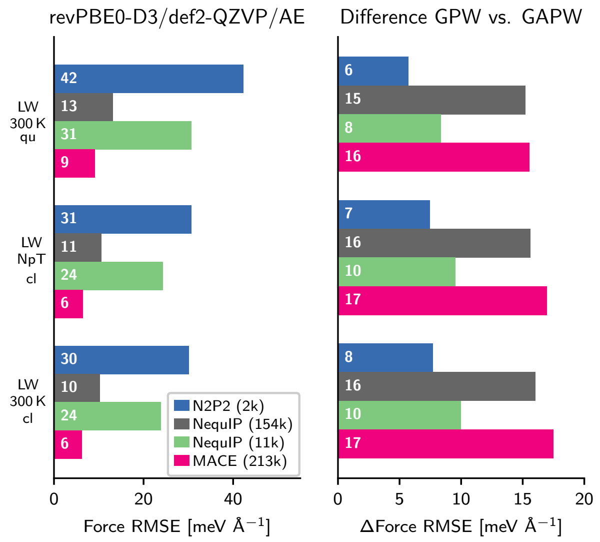

FIG. S11. The left panel shows the force RMSE of four different models architectures trained and tested on the revPBE0-D3/def2-QZVP/AE datasets. The right panel shows the difference in force RMSEs between revPBE0-D3/TZV2P/GTH models and revPBE0-D3/def2-QZVP/AE models when tested on their respective test sets. In the legend, the number in brackets is the number of trainable parameters in each model.

common settings, resulting in 213 000 parameters. The left panel of Figure S11 shows the errors of the four models on different liquid water test sets calculated with revPBE0-D3/def2-QZVP/AE. As expected, the modern message-passing models MACE and NequIP can reduce the force error considerably compared to N2P2 and the minimalist NequIP model performs worse than the baseline NequIP model. The right panel of Figure S11 shows the difference in test errors between the errors from revPBE0-D3/TZV2P/GTH models and revPBE0-D3/def2-QZVP/AE models tested on their respective test sets. As we have seen in Figure 1 of the main paper, the performance of the model is largely independent from the exact potential energy surface. Instead, the presence of errors in the datasets due to the egg box effect is determining the difference in test set errors of the revPBE0-D3/TZV2P/GTH and revPBE0-D3/def2-QZVP/AE models. It is clearly evident that this difference is larger for models, which have a high capacity and perform very well on the test set, than for models, which perform worse. We believe that the origin of this difference lies in the ability of a model to fit the noise in the training data stemming from the egg box effect. Low-capacity models such as N2P2 or the minimalist NequIP setup smooths out the noise

in the revPBE0-D3/TZV2P/GTH dataset, whereas high-capacity models are able to fit the training data so well that they fit some of the noise of the egg box effect.

REFERENCES

REFERENCES

[S1] C. Schran, K. Brezina, and O. Marsalek, J. Chem. Phys. **153** (2020), 10.1063/5.0016004.

[S2] S. Goedecker, M. Teter, and J. Hutter, Phys. Rev. B **54**, 1703 (1996).

[S3] T. Grindley and J. E. L. Jr., J. Chem. Phys. **54**, 3983 (1971).

[S4] E. Sanz, C. Vega, J. L. F. Abascal, and L. G. MacDowell, Phys. Rev. Lett. **92**, 255701 (2004).

[S5] M. Holz, S. R. Heil, and A. Sacco, Phys. Chem. Chem. Phys. **2**, 4740 (2000).

[S6] D. Kuryla, F. Berger, G. Csányi, and A. Michaelides, arxiv preprint (2025), 10.48550/arXiv.2510.19774.

[S7] B. Durham, M. I. J. Probert, and P. J. Hasnip, Electron. Struc. **7**, 025004 (2025).

[S8] G. Lippert, J. Hutter, and M. Parrinello, Mol. Phys. **92**, 477 (1997).

[S9] G. Lippert, J. Hutter, and M. Parrinello, Theor. Chem. Acc. **103**, 124 (1999).

[S10] A. Singraber, J. Behler, and C. Dellago, J. Chem. Theory Comput. **15**, 1827 (2019).

[S11] A. Singraber, T. Morawietz, J. Behler, and C. Dellago, J. Chem. Theory Comput. **15**, 3075 (2019).

[S12] S. Batzner, A. Musaelian, L. Sun, M. Geiger, J. P. Mailoa, M. Kornbluth, N. Molinari, T. E. Smidt, and B. Kozinsky, Nat. Commun. **13**, 2453 (2022).

[S13] I. Batatia, D. P. Kovacs, G. Simm, C. Ortner, and G. Csanyi, in *Advances in Neural Information Processing Systems*, Vol. 35 (2022) pp. 11423–11436.
